# Hybrid Nanostructured Compounds of Mo_2_C on Vertical Graphene Nanoflakes for a Highly Efficient Hydrogen Evolution Reaction

**DOI:** 10.1021/acsaem.3c00625

**Published:** 2023-05-19

**Authors:** Stefanos Chaitoglou, Roger Amade, Rogelio Ospina, Enric Bertran-Serra

**Affiliations:** † Department of Applied Physics, University of Barcelona, C/Martí i Franquès, 1, Barcelona, Catalunya 08028, Spain; ‡ ENPHOCAMAT Group, Institute of Nanoscience and Nanotechnology (IN2UB), University of Barcelona, C/Martí i Franquès, 1, Barcelona, Catalunya 08028, Spain; § Escuela de Física, Universidad Industrial de Santander, Carrera 27 calle 9 Ciudad Universitaria, Bucaramanga 68002, Colombia

**Keywords:** electrocatalysts, hydrogen evolution reaction, molybdenum carbide, graphene nanowall, graphene nanoflakes

## Abstract

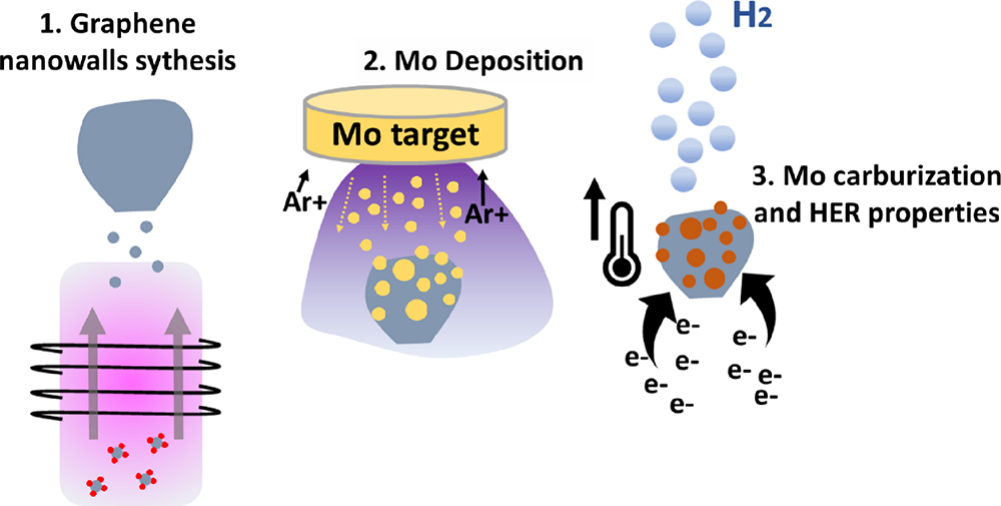

Organizing a post-fossil fuel economy requires the development of sustainable energy carriers. Hydrogen is expected to play a significant role as an alternative fuel as it is among the most efficient energy carriers. Therefore, nowadays, the demand for hydrogen production is increasing. Green hydrogen produced by water splitting produces zero carbon emissions but requires the use of expensive catalysts. Therefore, the demand for efficient and economical catalysts is constantly growing. Transition-metal carbides, and especially Mo_2_C, have attracted great attention from the scientific community since they are abundantly available and hold great promises for efficient performance toward the hydrogen evolution reaction (HER). This study presents a bottom-up approach for depositing Mo carbide nanostructures on vertical graphene nanowall templates via chemical vapor deposition, magnetron sputtering, and thermal annealing processes. Electrochemical results highlight the importance of adequate loading of graphene templates with the optimum amount of Mo carbides, controlled by both deposition and annealing time, to enrich the available active sites. The resulting compounds exhibit exceptional activities toward the HER in acidic media, requiring overpotentials of 82 mV at −10 mA/cm^2^ and demonstrating a Tafel slope of 56 mV/dec. The high double-layer capacitance and low charge transfer resistance of these Mo_2_C on GNW hybrid compounds are the main causes of the enhanced HER activity. This study is expected to pave the way for the design of hybrid nanostructures based on nanocatalyst deposition on three-dimensional graphene templates.

## 1 Introduction

The latest developments in fuel cell technology have increased expectations for the practical use of hydrogen as an energy carrier.^1−3^ Nevertheless, among the different hydrogen production approaches, namely, methane, natural gas, industrial carbon reforming, and water electrolysis, only the latest (water electrolysis) is considered climate-neutral and therefore complies with global efforts to reduce carbon emissions to net zero by 2050.^4^ Consequently, more research is being conducted on the development of efficient electrolyzers,^5^ especially on the use of electrocatalysts that facilitate the hydrogen evolution reaction (HER)^6−8^ The performance of noble metal–based catalysts (such as Pt and Pd) toward the HER remains unmatched;^9,10^ however, their high cost and scarcity limit their use in commercial applications.

Another class of materials that exhibits very promising electrocatalytic properties and is more accessible is transition-metal compounds^11−16^ and especially carbides (TMCs)^17,18^ like Mo_2_C, whose electrocatalytic performance in many cases approaches that of noble metals^19,20^ This electrocatalytic performance is afforded by its particular electronic structure. Incorporation of carbon atoms in the interstitial sites of the parent transition metal results in an increased metal–metal bond distance, leading to a contraction of the metal d-band and a higher density of states near the Fermi level. This explains the unique surface reactivity of Mo_2_C and most transition-metal carbide and nitride compounds.^21^ Therefore, research on novel approaches for synthesizing Mo_2_C-based compounds has increased since it exemplifies a realistic and economical electrocatalyst that remains chemically stable in acidic and alkaline media.^17^

Two approaches are used to further improve the electrocatalytic performance of Mo_2_C compounds: (i) enriching active sites of Mo_2_C by designing high-surface-area architectures and (ii) increasing electrode conductivity using highly conductive substrates. To increase active sites on Mo_2_C, Mo_2_C-based compounds have been synthesized in a variety of nanostructures, including nanowires,^22^ nanoparticles,^23^ nanobelts,^24^ and two-dimensional (2D) thin films.^25−27^ The conductivity of Mo_2_C is increased via hybridization with conductive materials such as graphene nanosheets,^28^ carbon nanotubes,^29^ and carbon foams.^30^ Theoretical calculations based on density functional theory have confirmed that the deposition of Mo_2_C on graphene structures reduces the free energy barriers of the HER mechanism by favoring the adsorption of H* and desorption of molecular hydrogen.^31,32^

**Scheme 1:**
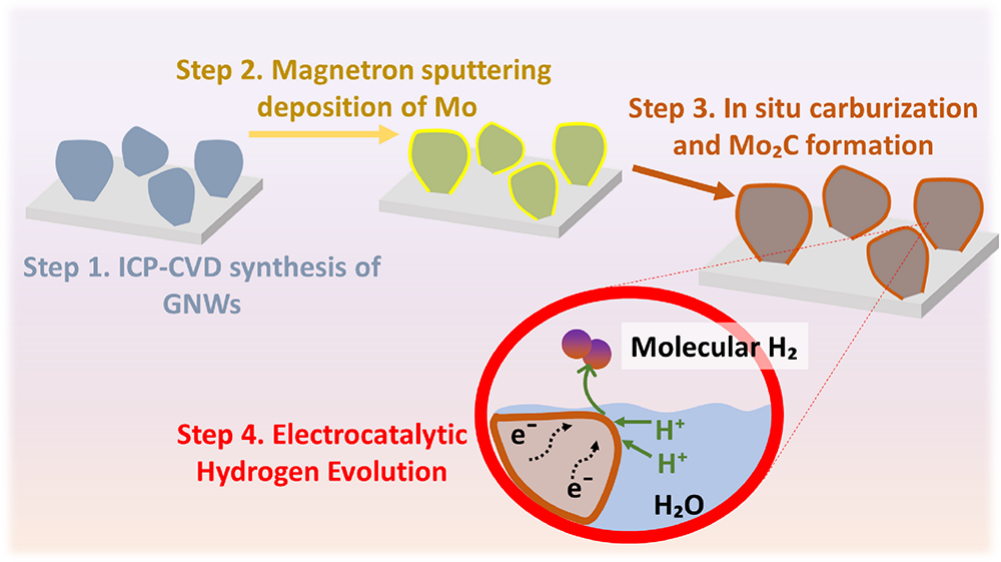
Schematic of the Deposition and Carburization of Nanostructured Mo Carbide on the GNW Template and Its Application in Electrochemical Hydrogen Evolution

This study reports an experimental approach for the preparation of Mo_2_C on graphene electrocatalysts, designed to address both aforementioned requisites (increase of active sites and enhancement of conductivity). For this purpose, the use of graphene nanowalls (GNWs) is a key feature in this study. GNWs, known also as vertical graphene flakes, are networks with a very high specific surface area of 1100 m^2^g^–1^,^33^ which is comparable to or higher than that of carbon nanotubes, a benchmark material used in energy-related applications that demand high active surface areas.^34^ The unique 2D structure of GNWs comprising dense networks of ultrathin walls with lengths in hundreds of nanometers affords them with a very high specific surface area. GNWs exhibit a high in-plane electrical conductivity^35^ that promotes their use in electrochemical applications. Mo_2_C nanostructures are deposited on current collectors by magnetron sputtering of Mo followed by in situ high-temperature annealing that facilitates Mo carburization. The resulting compounds can be directly applied as electrocatalysts in the HER (see process schematic in Scheme 1). Electrochemical analysis results make evident some important findings, related to the electrocatalytic performance of Mo_2_C on GNWs toward HER, that is (i) the benefit of using GNWs as a template, compared to a planar carbon substrate, (ii) the enhancement of performance after the carburization of Mo, compared to this of bare metallic Mo, and also (iii) the effect of annealing duration on the size of the formed Mo_2_C particles. Increasing the annealing time of Mo on GNWs results in the formation of larger Mo_2_C clusters, which exhibited poorer catalytic performance. On the other hand, smaller, optimized in terms of particle size, Mo_2_C compounds in the form of nanoparticles exhibit very efficient electrocatalytic performance toward the HER, accompanied by good durability.

## 2 Materials and Methods

### 2.1 Preparation and Physical Characterization of Nanostructured Electrodes

#### 2.1.1 Synthesis of GNWs

GNWs were deposited on Papyex flexible paper by inductively coupled plasma chemical vapor deposition (ICP-CVD). A detailed description of the synthesis process can be found in the literature.^36^ Herein, the Papyex substrate (∼35 × 50 mm) was cleaned using acetone and deionized (DI) water and dried using a N_2_ gun before inserting in the deposition reactor. A piece of graphite was used as a sample holder. The deposition reactor was an ICP-CVD system (13.56 MHz, power = 440 W) comprising a long quartz tube (Vidrasa S.A., Ripollet, Spain), a radio frequency (RF) resonator (homemade) for producing remote plasma, and a tubular oven (PID Eng & Tech S.L., Madrid, Spain). The Papyex sample was placed at a distance of 30 cm from the plasma zone and heated at 750 °C while the pressure in reactor was decreased to ∼10^–4^ mTorr using a turbomolecular pump. Briefly, first, a H_2_ plasma was applied for Papyex surface cleaning, at an RF power of 400 W in 400 mTorr of H_2_ pressure for 5 min. Then, the H_2_ flow was paused, and a CH_4_ plasma was produced under the same RF power and pressure conditions to initiate the GNW growth. GNW growth time was 30 min. The GNWs-on-Papyex sample was cooled to room temperature (20 °C approximately) under vacuum. Finally, a short-duration O_2_ plasma was applied at an RF power of 40 W in 400 mTorr for 30 s to enhance the hydrophilicity of the GNW surface. Then, the GNWS-on-Papyex sample was removed from the reactor.

#### 2.1.2 Deposition of Mo_2_C

The GNWs-on-Papyex sample was loaded in a sputtering chamber, which is coupled in line with a CVD oven. A 5 mm thick circular segment cut from a graphite bar was used as a sample holder. The whole magnetron sputtering and CVD oven system is a single unit, that is, there is no separation between the sputtering chamber and quartz oven. This system facilitates the deposition of metals via magnetron sputtering and consecutive thermal annealing under vacuum without exposure to the atmosphere. A detailed description of the system can be found in the literature studies.^37,38^ The pressure of the reactor was decreased to ∼10^–3^ mTorr using a turbomolecular pump. Mo was deposited on GNWs/Papyex by magnetron sputtering a high-purity Mo target (99.99%) at an RF power of 100 W in an Ar pressure of 70 mTorr for various deposition times. The deposition rate of Mo on Papyex was ∼10 nm/min, according to a prior calibration conducted on a glass substrate. Once Mo deposition was terminated, the Mo-on-GNW sample was transferred to the quartz tube oven. The oven was heated up to 950 °C while maintaining a pressure of 70 mTorr in a pure Ar atmosphere. Then, the Mo-on-GNW samples were annealed under the same atmospheric conditions for various times to carburize the Mo. The CVD oven system was cooled down to room temperature, and the Mo_2_C-on-GNW sample was extracted for characterization. For control samples of Mo on GNWs where no carburization took place, the sample was extracted from the magnetron sputtering chamber after Mo deposition.

#### 2.1.3 Physical Characterization

The morphology of the Mo_2_C-on-GNW samples was studied using scanning electron microscopy (SEM) (JEOL JSM-7001F, operated at 20 kV) and transmission electron microscopy (TEM) (JEOL 1010, operated at 200 kV). For observation on TEM, the nanostructures were transferred on a Cu grid by applying pressure with a cotton stick to remove from the growth substrate. SEM and TEM images were treated using ImageJ and Digital Micrograph software. X-ray photoelectron spectroscopy (XPS) was performed using a PHI 5500 Multi-Technique System (Physical Electronics, Chanhassen, MN, USA) with a monochromatic X-ray source (Al Kα line of 1486.6 eV energy and 350 W) placed perpendicular to the analyzer axis and calibrated using the Ag 3d_5/2_ line at a full width at half-maximum (FWHM) of 0.8 eV. The analyzed area was a circle with a diameter of 0.8 mm, and the selected resolution for the survey XPS spectra had a pass energy of 187.5 eV and 0.8 eV/step and the selected resolution for the elemental spectra had a pass energy of 11.75 eV and 0.1 eV/step. The vibrational modes of the Mo_2_C-on-GNW samples were studied using a Raman microscope (HR800, Lab-Ram; HORIBA France SAS, Palaiseau, France) with a 532 nm solid-state laser (laser power = 5 mW; diameter = ∼1 μm). For X-ray diffraction (XRD) measurements, a PANalytical XPert PRO MPD Bragg–Brentano powder diffractometer with a 240 mm radius was used. Samples were irradiated with a Co Kα radiation (λ = 1.789 Å) in a 2θ range from 4 to 99° with a step size of 0.017° and measuring time of 200 s per step.

### 2.2 Electrochemical Analyses

The electrochemical properties of the compounds were studied using a potentiostat/galvanostat (AutoLab, PGSTAT30, Eco Chemie B.V.). All experiments were performed at room temperature in a typical three-electrode cell. A Ag/AgCl electrode (an internal 3 M KCl solution) and a Pt electrode (purchased from Metrohm; the Pt tip was separated by porous glass to avoid dissolution into the electrolyte and sample contamination) were used as the reference and counter electrodes, respectively. The working electrode was nanostructured Mo_2_C deposited on the GNWs-on-Papyex or a bare Papyex substrate and was electrically connected to a power supply via a crocodile clip. The backside of the substrate was covered with insulating tape. Linear sweep voltammetry (LSV) was performed with a scan rate of 5 mV s^–1^ in a 0.5 M H_2_SO_4_ electrolyte. The surface area of the electrodes was always 1 cm^2^. LSV measurements were performed 10 times before recording the data to ensure stable performance of the electrode. The electrode endurance was evaluated via chronoamperometry using a constant bias of −0.082 V (vs reverse hydrogen electrode (RHE)). Charge transfer resistance was measured via electrochemical impedance spectroscopy (EIS) in the frequency range from 100 kHz to 1 Hz. Cyclic voltammetry (CV) was performed in the non-Faradaic voltage window of 0–0.7 V, where the compounds are electrochemically inactive, at a scan rate (*r_sc_*) of 10–100 mV/s. Capacitances were calculated from the slope from the straight line fit of the curve of *I*_max_ versus scan rates since 

All potentials were converted against the RHE using the Nernst law equation as follows:
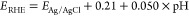
where *E*_RHE_ is the potential of the RHE and *E*_Ag/AgCl_ is the measured potential against the Ag/AgCl (3 M KCl) reference electrode. All electrodes were stored under ambient conditions and were characterized several days to weeks after electrode preparation. All electrochemical measurements were performed at room temperature.

## 3 Results and Discussion

### 3.1 Synthesis and Characterization of Nanostructured Mo_2_C on GNWs

**Figure 1:**
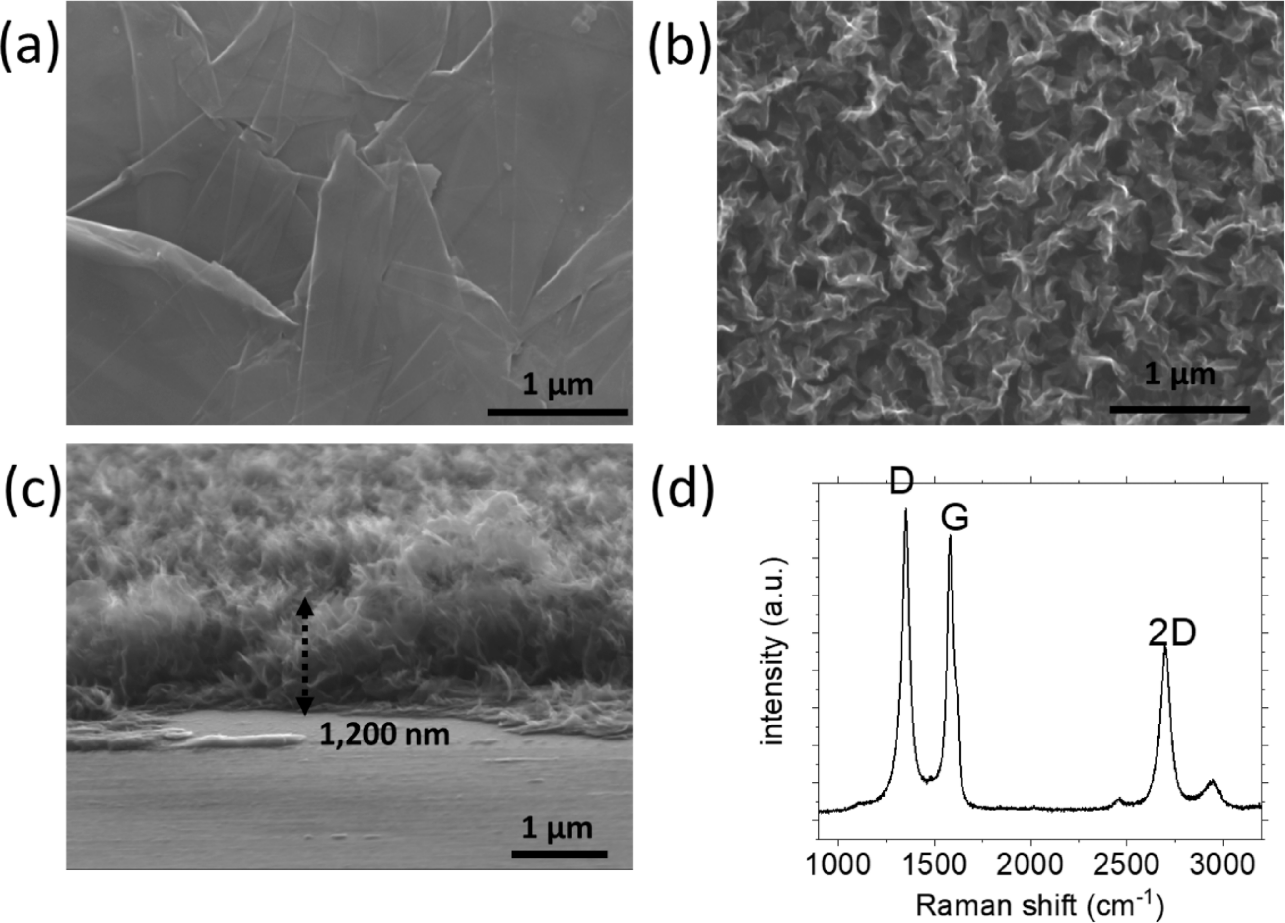
SEM images of the (a) bare Papyex surface (top view), (b) GNWs deposited on Papyex (top view), and (c) GNWs deposited on Papyex (side view). (d) Raman spectrum of GNWs deposited on Papyex. The D, G, and 2D bands of graphene are noted in the figure.

**Figure 2:**
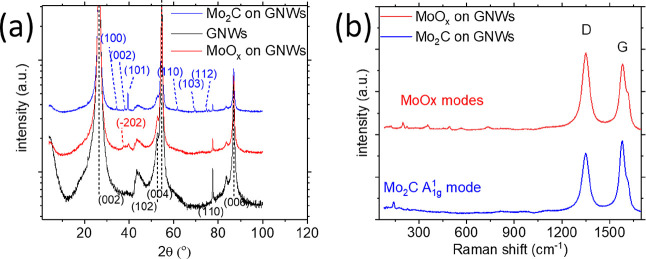
(a) XRD patterns of bare GNWs (black curve), insufficiently carburized MoO_*x*_ (red curve), and Mo_2_C (blue curve) deposited on the GNWs substrate. The crystallographic orientation marked in black correspond to graphite, those marked in red correspond to Mo_2_ and those marked in blue correspond to Mo_2_C. (b) Raman spectra of insufficiently carburized MoO_*x*_ (red curve) and Mo_2_C (blue curve) deposited on the GNW substrate. The Mo compound modes and D and G mode of graphene are noted in the figure.

GNWs were deposited on Papyex paper. SEM images of the substrate surface before and after GNW deposition are shown in Figure 1a,b. Detailed characterization of this material is reported in the literature.^36^ Papyex paper is composed of graphitic crystals with a nonpreferred orientation, exhibits a high specific absorption surface area, and is chemically stable.^39^ The main morphological features of the GNWs are their length of 150–250 nm and height of ∼1200 nm (Figure 1c). Raman spectra of the GNWs show that the thickness of the GNWs is 7–8 atomic layers [the FWHM value of the 2D peak (centered at ∼2690 cm^–1^) is measured to be 75 cm^–1^ and is used to estimate the number of layers of GNWs (Figure 1d)^40^]. Carburization was performed by first depositing Mo on the GNWs-on-Papyex substrate and annealing at a high temperature (∼950 °C) for a few minutes to get carburized. No additional carbon precursor was introduced; therefore, carburization occurs owing to the migration of C species (probably deposited amorphous C or C attached at defective sites) from the GNWs to Mo and reaction with Mo. More results and aspects of carburization through carbon migration will be discussed later in the manuscript. Carburization was verified by XRD and Raman and XPS spectroscopy. Figure 2a shows XRD patterns of bare GNWs on Papyex (black line), Mo on GNWs (blue line), and Mo_2_C on GNWs(red line). The various crystallographic orientations are noted in the figure and compared to data from databases (Figure S1). The XRD pattern of the Papyex paper (Figure 2a) exhibits various diffraction peaks, indicating that it has a polycrystalline nature. As reported in the literature and verified by XRD studies, the XRD pattern of bare GNWs exhibits a diffraction peak at 30.35°,^36^ coinciding with the peak of Papyex. The XRD pattern of the Mo-on-GNW sample that has not been exposed at annealing exhibits additional diffraction peaks at 37.40° and 39.77° corresponding to the (−202) plane of MoO_2_ and the (102) plane of MoO_3_, respectively.^41,42^ The XRD pattern of efficiently carburized Mo exhibits diffraction peaks at ∼34.44°, 37.95°, 39.45°, 61.61°, 69.57°, and 74.69°, corresponding to the (100), (002), (101), (110), (103), and (112) planes of orthorhombic a-Mo_2_C, respectively.^43^ Many of these faces are observed in TEM images, as will be shown below. Raman spectra confirmed the observations regarding formation of Mo carbides and oxides presented above. Figure 2b shows the Raman spectrum of poorly carburized Mo (blue line). The various Raman bands observed in the range 250–600 cm^–1^ reveal the formation of Mo oxides Mo_2_ and Mo_3_, respectively.^44^ Poorly carburized Mo films (and consequently oxidized once exposed to air) are formed at carburization temperatures of 900 °C or below, neither when applying to anneal in the Ar atmosphere nor in methane or acetylene atmosphere. On the other hand, well-carburized films (Figure 2b, blue line) exhibit an intense Raman band at ∼143 cm^–1^, attributed to a-Mo_2_C.^27^ The positions of the graphene and Mo carbide and oxide bands are tabulated in Table 1.

**Table 1:** Raman Modes of Graphene and Mo Carbides and Oxides and Their Position

Raman mode	position (cm^–1^)
graphene D	1347
graphene G	1581
graphene 2D	2690
Mo_2_C *A*_1*g*_^1^	143
MoO_x_	250–600

Various control experiments were performed to determine the optimum amount of deposited Mo. The deposition time used in this study was validated by studying the catalytic activity of bare Mo deposited on GNWs electrodes. The results demonstrate that the highest catalytic activity was achieved when the Mo deposition time was 150 s. Samples prepared using shorter and longer deposition times exhibit reduced catalytic activities, most probably because of insufficient catalyst loading or formation of larger clusters that reduce surface areas (Figure S2).

**Figure 3:**
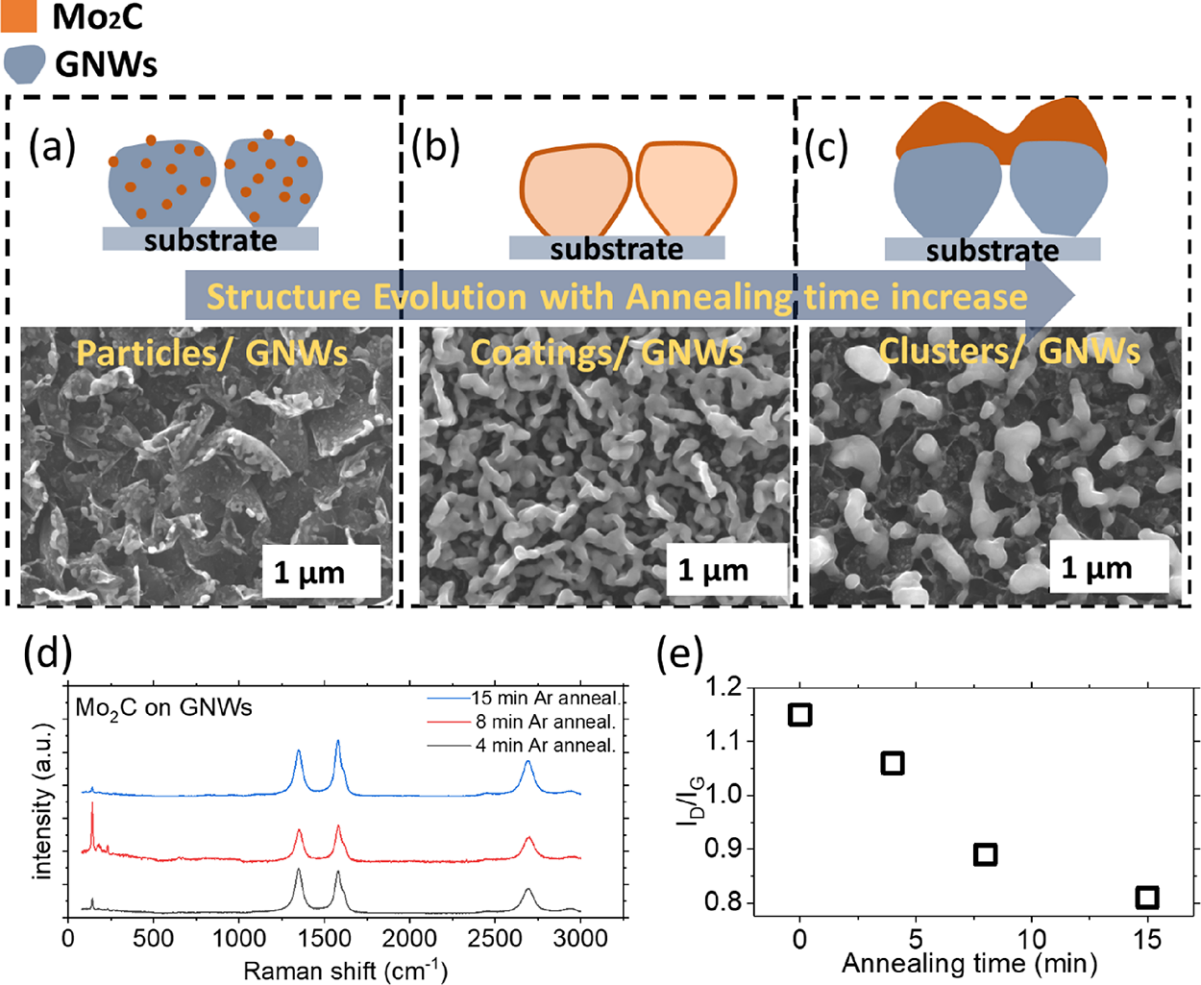
SEM images (lower) of Mo_2_C formed on GNWs after (a) 4 min, (b) 8 min, and (c) 15 min of annealing and illustrations of the resulting nanostructures (upper). (d) Raman spectra of Mo_2_C formed on GNWs after 4, 8, and 15 min of annealing. (e) Graph of the *I*_D_/*I*_G_ ratio as a function of annealing time.

Different annealing times were applied for the carburization steps. The morphology and crystal quality of the resulting nanostructures were studied via SEM and Raman spectroscopy. Digital images of the -on-Papyex and the Mo_2_C-on-GNW sample are shown in Figure S3a,b. SEM images in Figure 3 show Mo nanoparticles deposited on GNWs and annealed for 4 min (Figure 3a), 8 min (Figure 3b), and 15 min (Figure 3c) in an Ar atmosphere. In all three cases, Raman spectra resemble the fingerprint of Mo_2_C (Figure 3d). The same amount of Mo is deposited on all samples; however, the resulting nanostructures greatly vary. For the sample with the shortest annealing time (Figure 3a), small Mo_2_C nanoparticles are formed, deposited on the GNWs. Extensive characterization of these nanoparticles by TEM will be discussed in the following section. For the sample with a medium annealing time (Figure 3b), Mo_2_C with larger structures was formed, which were deposited as continuous coatings on the GNW surface because of Mo_2_C particles agglomerating and forming larger agglomerates. The coalescence of metallic nanoparticles during high-temperature thermal annealing is a well-known phenomenon that often alters their properties.^45^ For the sample with a longer annealing time (Figure 3c), larger clusters are formed as a result of the nanoparticles ripening (see also agglomerated Mo distribution in EDS elemental mapping shown in Figure S4). An illustration of the various Mo_2_C on GNWs nanostructures is shown in Figure 3a–c as a guide for the understanding of their morphologies. The *I*_D_/*I*_G_ ratio was calculated for each Raman spectrum. The *I*_D_/*I*_G_ ratio indicates the amount of crystal defects in the graphene lattices,^46^ and it is widely used to characterize the crystal quality.^47,48^ At the same time, these defective sites may favor the bonding of the Mo_2_C structures on the GNWs. As the annealing time increases, the *I*_D_/*I*_G_ ratio decreases (Figure 3d,e), indicating a decrease in the number of defects on GNWs, supporting the hypothesis that amorphous C species present on GNWs migrate and carburize Mo particles during annealing. In the control experiment, bare GNWs on Papyex were annealed at 950 °C for 15 min in the absence of any additional Mo. Raman spectra before and after annealing were identical and the *I*_D_/*I*_G_ ratio is the same, indicating that in the absence of Mo, annealing does not change the graphene nanostructure (Figure S5). Moreover, even in the presence of Mo, if the annealing temperature is not sufficient to provoke carburization, the Raman spectrum of GNWs remains unchanged, as shown in Figure 2b (red line) where the annealing temperature is 900 °C or less. The resulting compound is MoO_*x*_, and the *I*_D_/*I*_G_ ratio remains the same as that of the fresh GNW sample (Raman spectrum in Figure 1d).

As explained above, carburization was performed in a pure Ar atmosphere without any additional carbon precursor. Nevertheless, control experiments were performed, where CH_4_ and C_2_H_2_ were introduced in the chamber during the annealing step to serve as C precursors. The GNWs were etched during carburization when CH_4_ and C_2_H_2_ gases were used. SEM images show the removal of the GNWs from the Papyex surface followed by the deposition of Mo_2_C particles (Figure S6a). Raman spectra of the sample before (Figure S6b, red line) and after Mo_2_C deposition (Figure S6b, black line) reveal the deterioration in the crystal quality of the GNWs owing to the annealing process. The decrease in the crystal quality is most probably attributed to the high concentration of H_2_, originating from the precursor gas, that aggressively etches the GNWs. While C species sufficiently react with Mo and carburize it, the resulting nanostructures exhibit a reduced volume and surface area, making them inappropriate for catalytic applications.

**Figure 4:**
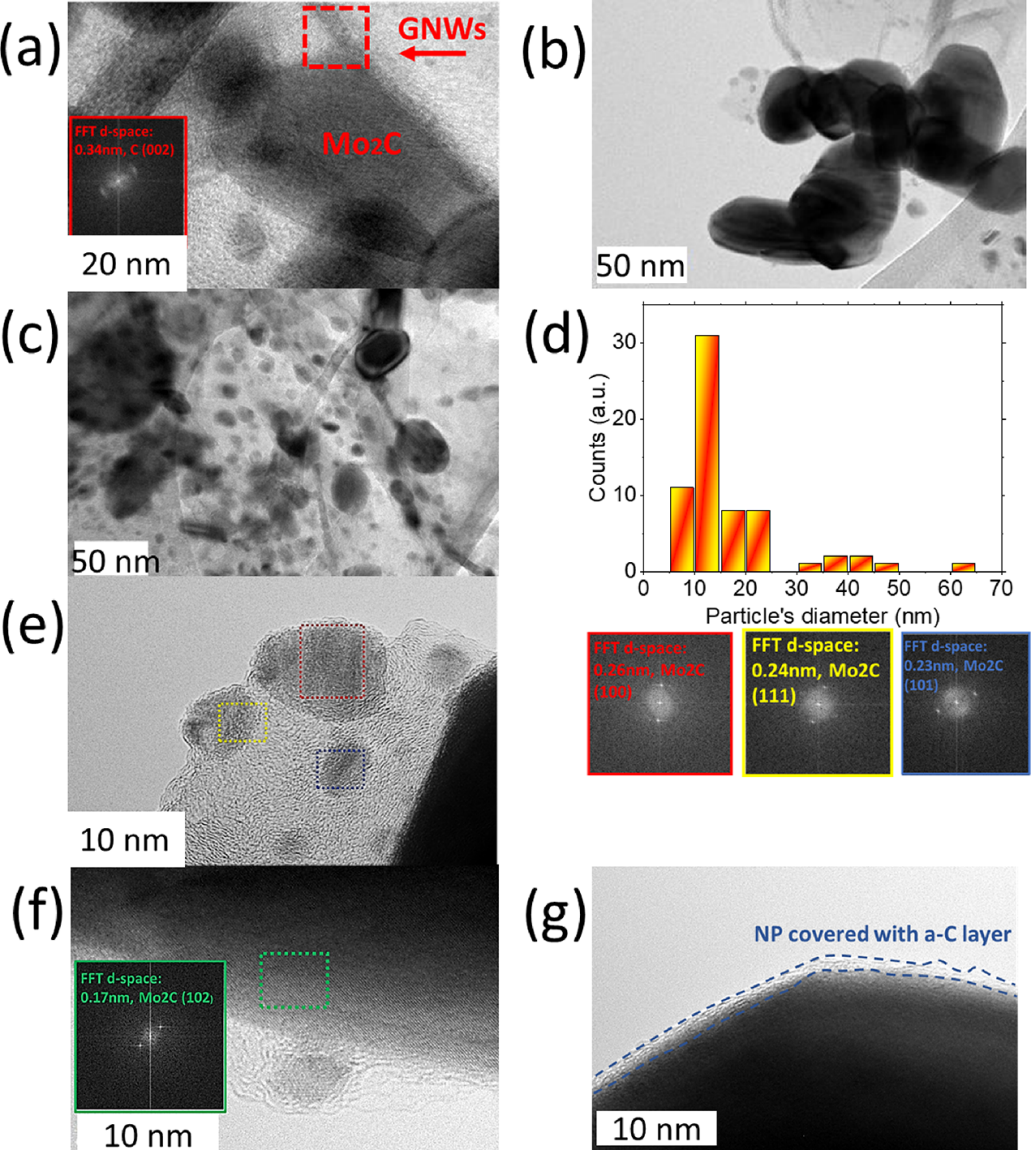
TEM images of (a) Mo_2_C nanoparticle anchored on a GNW, (b) agglomerated Mo_2_C nanoparticles, and (c) distribution of Mo_2_C nanoparticles on a graphene sheet. (d) Size distribution histogram of the Mo_2_C nanoparticles. (e) High-resolution TEM images and (f) corresponding FFTs of Mo_2_C nanoparticles and corresponding FFTs. (g) TEM image of a Mo_2_C particle with a carbon shell on the surface.

The TEM image of the sample in Figure 3a shows the crystal structure and dimensions of Mo_2_C nanoparticles deposited on GNWs. The results are presented in Figure 4. Figure 4a shows the anchoring of a Mo_2_C particle on a GNW edge. Fast Fourier transform (FFT) analysis exhibits that the GNW structure has a lattice spacing of 0.34 nm, in agreement with the lattice spacing of graphene.^49^Figure 4b shows the formation of agglomerated particles anchored on a GNW sheet. Figure 4c shows the homogeneous and dense deposition of particles on the graphene sheet. This finding is important since it reveals that the total active surface area of Mo_2_C in this sample is probably larger than that in the samples obtained after longer annealing periods. Thus, this may explain the enhanced electrocatalytic activity of this sample, as discussed later in the article. The size distribution histogram is shown in Figure 4d. A majority of particles have diameters of ∼10–20 nm; however, a second smaller group of particles with diameters of ∼30–50 nm is the result of the previously showed agglomerations. Figure 4e shows the high-resolution images of the nanoparticles, in which various planes can be distinguished. The FFT reveals the presence of (111), (100), (101), and (102) planes (Figure 4f).^50,51^ Even larger particles, with diameters ≥40 nm, appear to be single crystals (Figure S7). All planes observed by TEM have been identified in the XRD pattern as well. Additionally, the formation of a thin C shell with a thickness ∼2–3 nm on the surface of the Mo_2_C nanoparticles was also observed. Such shells are often observed when carbide materials are prepared by heat treatment using carbon-containing gas precursors.^49^ These shells decrease the catalytic activity and improve the stability of nanoparticles.^52^ Noteworthily, no MoO_x_ planes were observed in the TEM images, neither in the core nor in the outer planes of the particles, in line with the XRD results. Considering this aspect, the thin C shell is expected to play an important role. As suggested in the literature, the shell can suppress surface oxidation by acting as a mechanical barrier that blocks the volume expansion attributed to oxidation. This may explain why Mo_2_C maintains its rich catalytic activity long term. However, the C shell is not really a chemical barrier since molecules can still penetrate it and reach catalytically active sites on the Mo carbide surface.^50^

**Figure 5:**
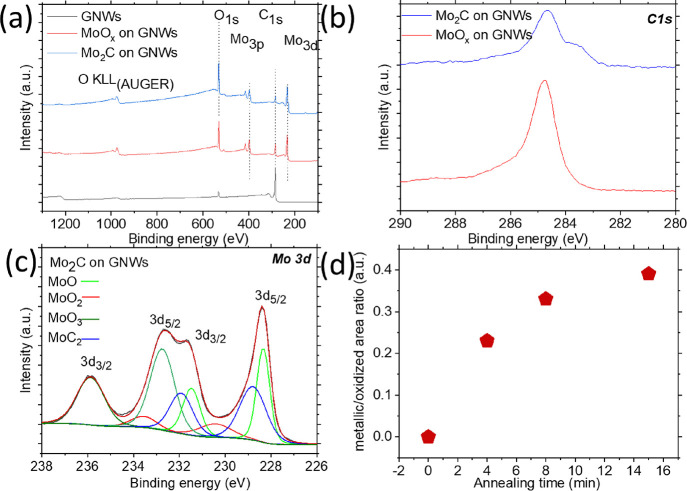
XPS (a) survey spectra of samples, (b) C_1s_ spectra before (red) and after (blue) Mo carburization, and (c) Mo_3d_ spectra after Mo carburization. (d) Graph with the metallic/oxidized area ratio with respect to the annealing time, calculated after fitting the Mo_3d_ peak components.

The surface states of the Mo compounds were characterized by XPS. The survey spectra are shown in Figure 5a. All peaks are assigned to signals from C, Mo, and O, confirming the absence of any surface contamination on the samples. Figure 5b shows the C 1s peak of the compounds, raising some notable observations. The peak corresponding to the C–C bond is centered at 284.8 eV and is attributed to sp^2^ configurations present in the GNW structures (red curve). The C 1s peak remains unchanged after Mo deposition (Figure S8), revealing that the deposited Mo does not react with the GNW template. After carburization, a second peak appears at 283.8 eV (blue curve). This second peak, centered to a lower binding energy, is caused by the reaction between C atoms and less electronegative Mo atoms, forming the C–Mo bond (Figure S9). Additionally, the deconvolution of the C 1s peak reveals a minor contribution from other components, specifically C–O, C–OH, C=O, O–C=O, and O-C-OH, that have previously identified as present in GNWs.^36,49^Figure 5c shows the Mo 3d peaks of the carburized Mo carburized compounds. The Mo 3d peak of the as-deposited Mo is depicted in Figure S10. The Mo peaks can be divided into four doublets. In the as-deposited Mo sample (Figure 5c, bottom spectrum), which has not undergone carburization, strong surface oxidation is evident, as indicated by the peaks at 232.9 and 236.1 eV corresponding to MoO_2_ and MoO_3_, respectively. Peaks attributed to carburized Mo are absent.^23,53^ At high temperatures, C atoms displace O atoms and react with Mo atoms to form carbide compounds. As a result, the carburized Mo sample exhibits additional peaks at 228.5 and 231.9 eV, corresponding to the reaction between Mo and C and the presence of MoO,^54,55^ respectively, as is evident from the deconvoluted peaks (Figure 5c, top spectrum). However, the peaks related to surface oxidation are still distinct (same spectrum as before). With increasing annealing time, the area ratio between carburized and oxidized Mo increases, as revealed by the deconvolution of the Mo 3d peak components (Figure 5d and Figures S11a–c). Specifically, there is an increase in the metallic Mo components at 229 and 232.6 eV, attributed to carburization. This is associated with the formation of larger agglomerated Mo carbides, wherein the core maintains its metallic character, while the surface is oxidized after exposure to the atmosphere.

Nevertheless, by observing these results and having in mind that Raman spectroscopy and XRD characterization show no evidence of Mo oxidation on the carburized compounds, therefore, oxidation is only superficial and does not suppress the catalytic activity of Mo carbides. The aforementioned role of the carbon shell becomes evident here since the C shell mechanically confines the volume expansion of carbide catalysts and hinders their oxidation.

### 3.2 Application in the Electrocatalytic HER

**Figure 6:**
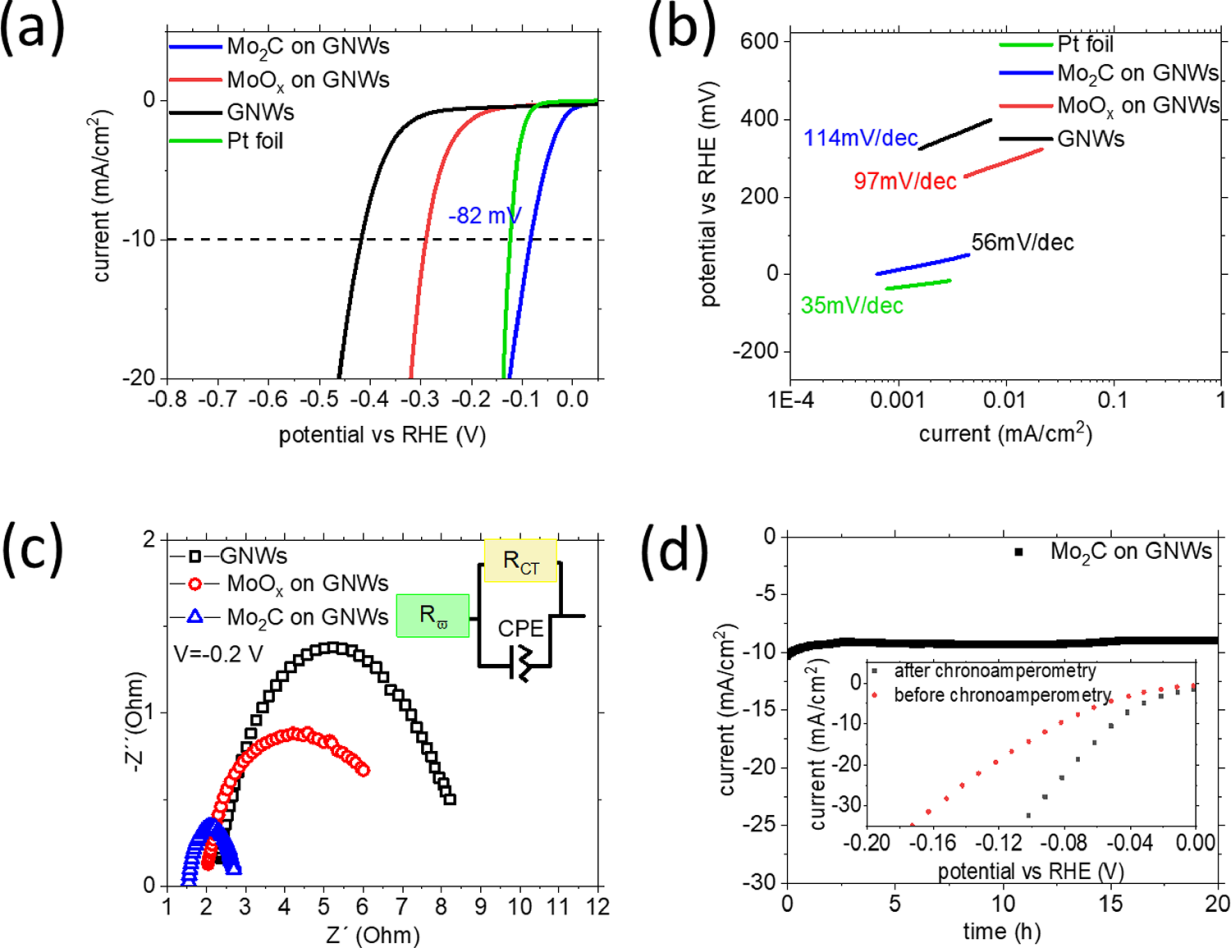
(a) LSV curves of Mo carbide (blue), as-deposited Mo (red), and bare GNWs on Papyex. (b) Tafel slopes produced from the LSV curves. (c) EIS curves of the electrodes. (d) Chronoamperometry test during 20 h under −85 mV continuous bias and (inset) LSV curves comparing the catalytic activity before and after the endurance test.

The electrocatalytic properties of Mo_2_C nanostructures toward the HER were evaluated by LSV and impedance spectroscopy. Figure 6a shows the polarization curves of bare GNWs on Papyex (black line), as-deposited Mo on GNWs (red line), and carburized Mo_2_C on GNWs (blue line). Results show the enhancement in the performance of the carburized Mo structures. The bare GNWs-on-Papyex electrode shows the worst performance and requires −419 mV to generate 10 mA/cm^2^. Even though graphene materials are widely considered as electrocatalytically inactive, there are experimental and theoretical proofs that denote the defective edges of GNWs as active sites toward the HER.^56^ The as-deposited Mo on GNWs exhibits an onset potential of −130 mV and an overpotential of −293 mV for the generation of −10 mA/cm^2^. The 4 min-annealed carburized Mo exhibits an onset potential of −21 mV (for production of −1 mA/cm^2^) and an overpotential of −82 mV for the generation of −10 mA/cm^2^. This very high activity exhibits that the efficiency of the present Mo_2_C on GNW compounds can be fairly compared with that of Pt-foil electrodes (Figure 6a, green line). The very efficient activity of these Mo_2_C nanostructures is a result of their hybridization with the GNW template, which offers formation of a densely distributed ensemble of small crystal nanoparticles with abundant active sites. Figure 6b shows the Tafel slopes of all the electrodes. The bare GNWs-on-Papyex (black line) sample shows a Tafel slope of 114 mV/dec, the as-deposited Mo-on-GNW sample shows a Tafel slope of 97 mV/dec, and the Mo_2_C-on-GNW sample a Tafel slope of 56 mV/dec, revealing that the faster reaction kinetics occurs in the carburized electrode. EIS was used to study the interfacial charge transfer kinetics (Figure 6c). The Nyquist plot is fitted with a Randle’s circuit to extract the series and charge transfer resistances (Table 2). As expected, a dramatic drop in the charge transfer *R*_CT_ and series resistance (*R*_ω_) is observed between the bare GNWs (6.70 Ω), Mo deposited on GNWs (5.18 Ω), and Mo_2_C on GNWs (1.25 Ω), which may be attributed to high-temperature annealing in which Mo has been exposed. These findings indicate improved charge transfer dynamics at the electrode–electrolyte interface for the Mo_2_C-on-GNW electrode.

**Table 2:** Equivalent Circuit Parameters Obtained from Fitting the EIS Data

electrode material	*R*_ω_ (Ω)	*R*_ct_ (Ω)	*Q*_o_ (Ω^–1^s^n^/n)
GNWS	2	6.77	0.0003/0.50
MoO_x_ on GNWS	1.77	5.18	0.0008/0.43
Mo_2_C on GNWS	1.5	1.25	0.0013/0.64

A chronoamperometry test was used on the Mo_2_C (prepared by 4 min Ar annealing)-on-GNW electrode by applying a stable overpotential of −82 mV. A flat current response of −10 mA/cm^2^ was recorded for a period of 20 h, showing excellent stability with no apparent activity loss (Figure 6d). LSV curves before and after the chronoamperometry test were compared and showed that the performance of the electrode improved once the test was terminated (inset in Figure 6d). Specifically, there is a 27 mV decrease in the required overpotential to produce −10 mA/cm^2^ (from −82 to −55 mV). In the literature, such a reduction is attributed to the reduction of surface hydroxides during the initial hydrogen evolution.^57^

Additional chronoamperometry tests were performed on the Mo_2_C (prepared by 8 min Ar annealing)-on-GNW sample at a higher overpotential of −200 mV, and a flat current response of −25 mA/cm^2^ for 1 h was recorded (Figure S12a). The electrode was then characterized by SEM and EDS to evaluate its chemical and structural durability. EDS analysis showed no contamination of the sample (Figure S12b) since the spectra before (black line) and after (red line) the chronoamperometry test were almost identical. In the post-test spectrum, a small quantity of S is detected, probably originating from the electrolyte. SEM analysis shows no evidence of structural degradation after the durability test (Figure S12c). Furthermore, XRD analysis of the sample after the durability test shows no changes in the crystal structure or any oxidation-related degradation (Figure S12d). These results confirm the remarkable stability of Mo_2_C compounds in acidic electrolytes and under overpotential biases for up to tenths of hours, as previously reported.^58,59^ These present results add to the growing body of evidence for the promising potential of this class of materials for electrocatalysis.

**Figure 7:**
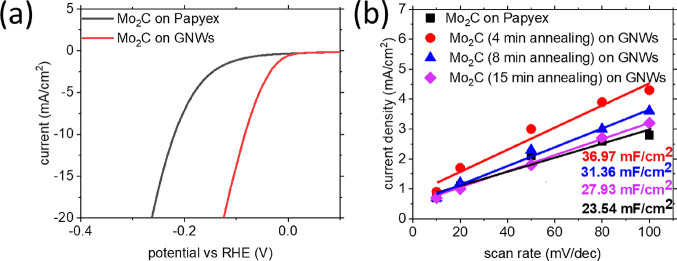
(a) LSV curves of Mo carbide particles deposited on the GNW template (red, 4 min of annealing) and a planar Mo carbide film deposited on Papyex. (b) Plot of current density with respect to scan rates applied during CV for planar and nanostructured Mo_2_C compounds, prepared under varying annealing times.

To provide further evidence regarding the beneficial effect of Mo_2_C deposition on GNWs and investigate the origin of enhanced HER, the performance of the electrode is compared to that of a planar Mo_2_C film deposited directly on a Papyex substrate. The latest was synthesized under the same conditions, and CH_4_ was used as the C precursor. The deposition time of Mo and thermal treatment time for carburization are the same. The absence of any oxidation peak in the Raman spectra of Mo_2_C show that the planar film is completely carburized (Figure S13). The SEM image shows the formation of a continuous film with nanostructured features, similar to the morphology of the underlying Papyex substrate (Figure S14). LSV curves are shown in Figure 7a. Results show a reduction of 138 mV on the overpotential values required to produce −10 mA/cm^2^ between the planar Mo carbide film (−223 mV) and the nanostructured Mo_2_C deposited on the GNWs template (−82 mV). CV was performed at different scan rates on the two electrodes to calculate the double-layer capacitance. The results are shown in Figure 7b. A ∼50% increase in the capacitance of the Mo_2_C particles on GNWs (36.97 mF/cm^2^) compared to that of planar Mo_2_C on Papyex (23.54 mF/cm^2^) was measured. Mo_2_C compounds prepared after 8 and 15 min of thermal annealing exhibit capacitances of 31.36 and 27.93 mF/cm^2^, respectively. The respective CV graphs are shown in Figure S15a–d.

**Figure 8:**
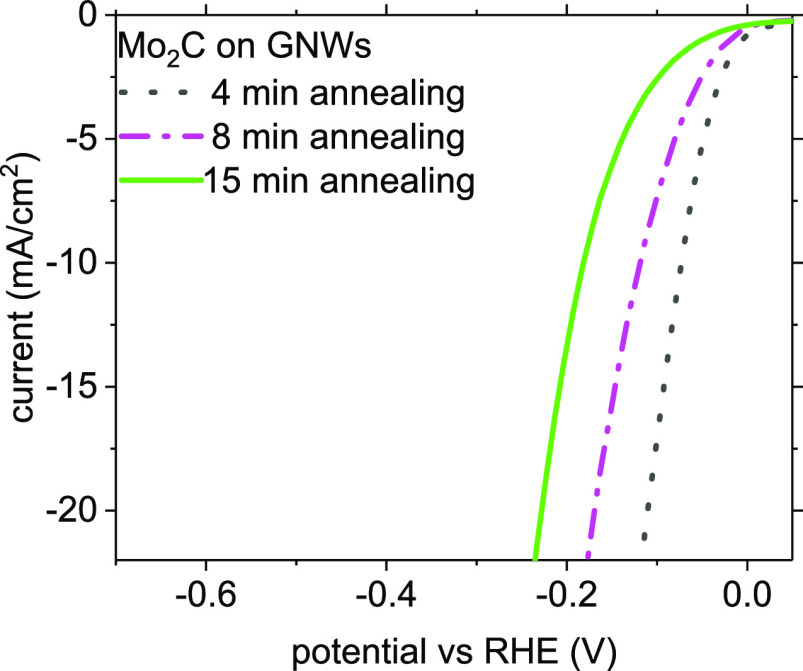
LSV curves of carburized Mo nanostructures deposited on GNWs under varying thermal annealing periods of 4 min (black curve), 8 min (pink curve), and 15 min (green curve).

For comparison, the double-layer capacitance of bare GNWs is calculated to be only 4 mF/cm^2^ (CV and current density/scan rate graphs are available at Figure S16a,b). Additionally, the LSV curves of the Mo_2_C nanostructures obtained under varying annealing times are compared and discussed. The three different kinds of nanostructures are shown in Figure 2a–c. The corresponding LSV curves are presented in Figure 8. The overpotential values for the production of −10 mA/cm^2^ are −82 mV for carburized Mo nanoparticles, −122 mV for carburized Mo coatings, and −185 mV for carburized Mo agglomerates, respectively. This evidence further supports the superior catalytic activity of the smaller Mo_2_C particles to that of the larger structures.

Additionally, it is evident that the catalytic activity of the nanostructured Mo_2_C is related to the enhanced capacitance since a more available active surface implies the presence of more catalytically available Mo active sites. Compounds annealed for a shorter time exhibit a higher capacitance and better catalytic activity. The above argument is additionally supported by the comparison between the planar Mo_2_C and the hybrid nanostructured Mo_2_C on GNWs. These findings strengthen the argument developed throughout the present study, which relates the excellent electrocatalytic activity toward HER to the abundance of active sites present in the nanometric Mo_2_C particles.

**Figure 9:**
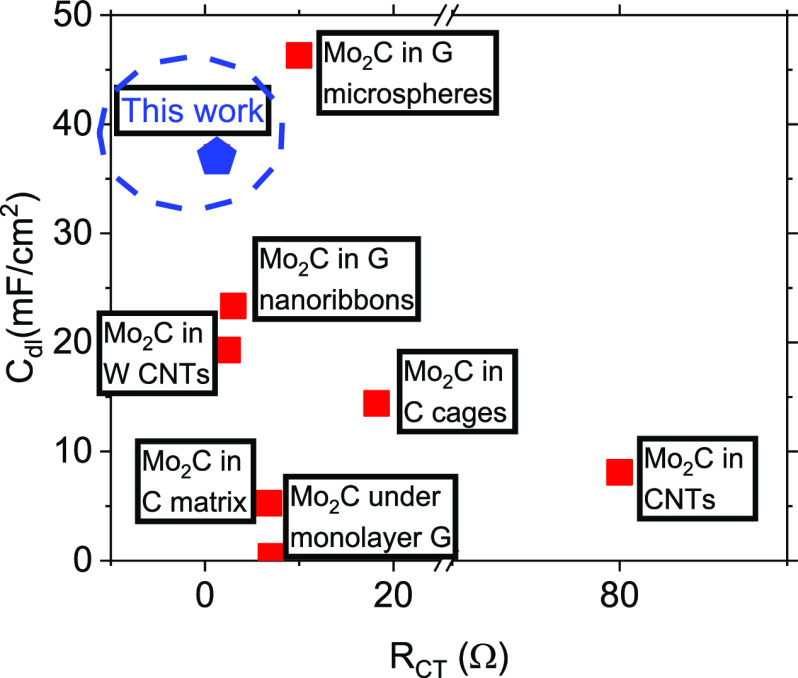
Comparison of double-layer capacitance and charge transfer resistance values between best Mo_2_C combined with graphene compounds HER catalysts found in the literature and this study.

To underline the excellent HER properties of the as-synthesized hybrid Mo_2_C-on-GNW electrodes, the overpotential values (*j* = 10 mA/cm^2^) of various nanostructured Mo_2_C on graphene hybrid compounds were compared. The properties of the compounds are superior to most reported molybdenum carbide-graphene catalysts in 0.5 M H_2_SO_4_, such as graphene on carburized Mo foil (*n*_10_ = 270 mV), graphene on 2D Mo_2_C (*n*_10_ = 236 mV), Mo_2_C in carbon cages (*n*_10_ = 198 mV), Mo_2_C on whisker carbon nanotubes (W-CNTs) (*n*_10_ = 187 mV), Mo_2_C in a carbon matrix (*n*_10_ = 182 mV), Mo_2_C on CNTs (*n*_10_ = 160 mV), and Mo_2_C on graphene ribbons (*n*_10_ = 150 mV), and only inferior to Mo_2_C in graphene microspheres (Table 3).^23,43,58−63^ Comparison of the double-layer capacitance and charge transfer resistance values between the various electrodes provides insights regarding the superior catalytic activity of the present compounds. Apparently, the (4 min annealed) Mo_2_C-on-GNW compounds exhibit the smallest charge transfer resistance and second highest double-layer capacitance values between the best Mo_2_C combined with graphene HER catalysts found in the literature (Figure 9). Since the HER activity is directly related to these properties, the reasons behind the excellent performance of the present electrodes become evident.

**Table 3:** Comparison of Overpotential Values Required to Produce 10 mA/cm^2^ in Acidic Medium for Various Mo_2_C on Carbon Compounds

compound material	overpotential @ 10 mA (mV) vs RHE	ref
Mo_2_C in graphene microspheres	70	(23)
Mo_2_C on GNWs	82	present work
Mo_2_C on graphene nanoribbons	150	(63)
Mo_2_C on CNTs	160	(59)
Mo_2_C in carbon nanocages	198	(61)
Mo_2_C on whisker CNTs	187	(62)
2D Mo_2_C on single layer graphene	236	(58)
Mo_2_C under single layer graphene	270	(43)
Mo_2_C in carbon matrix	182	(60)

## 4 Conclusions

This study reports results on the deposition of Mo via magnetron sputtering on GNW templates previously grown on Papyex flexible paper followed by in situ carburization through thermal annealing. The GNWs serve as the growth template and carbon source for carbide formation. At the same time, the abundant defects on the graphene lattice favor the bonding of the Mo_2_C nanostructures. Results show that depending on the annealing time, Mo carbide morphology greatly varies from initially formed particles of nanometric diameters to larger agglomerations after longer annealing treatments. Therefore, the specific surface area greatly varies, affecting the available active sites. Consequently, the electrocatalytic activity of the structures toward the HER varies as well. Structural and electrochemical characterization shows that smaller particles densely deposited on the graphene sheets are those with the better catalytic activity, owing to the abundance of active sites. Specifically, this nanostructured Mo_2_C-on-GNW electrode exhibits a smaller Tafel slope than planar Mo_2_C and pristine GNWs (56, 120, and 113 mV/dec, respectively) and a reduced required overpotential to produce a 10 mA/cm^2^ current density (82, 220, and 410 mV, respectively).^36^ Moreover, it outperforms the activity of the larger Mo_2_C-on-GNW electrodes. Indeed, the activity of these particles competes with Pt catalysts and is one of the highest reported for Mo_2_C structures reported in the literature.^50,58^ The findings and discussion presented in this study provide new insights into the preparation of nanostructured Mo_2_C on graphene nanoflake templates for application in electrocatalysis.

## Supporting Information Available

Data of the JCPDS patterns as guide for XRD results; LSV curves of Mo deposited on GNWs for various times; digital images of the as-prepared samples; EDS maps of C and Mo; Raman spectra of GNWs before and after high-temperature annealing; SEM image of Mo on GNWs after carburization in a CH_4_ atmosphere; TEM image of Mo_2_C particles; XPS spectrum of the C 1s peak before and after Mo deposition; XPS spectrum of the C 1s peak before and after carburization; XPS spectrum of the Mo 3d peak for various annealing times; chronoamperometry test results and sample characterization after the test; Raman spectrum of Mo_2_C on Papyex; SEM image of Mo_2_C on Papyex; CV graphs of Mo_2_C samples deposited on GNWs; CV graphs of bare GNWs (PDF)
