# Supplementary material for: Hybrid Nanostructured Compounds of Mo2C on Vertical Graphene Nanoflakes for a Highly Efficient Hydrogen Evolution Reaction
Source: ACS Appl Energy Mater. 2023 May 19;6(11):6120–31. doi: 10.1021/acsaem.3c00625 (PMC10265661; doi:10.1021/acsaem.3c00625)
Supplement: Supplementary file 1 — ae3c00625_si_001.pdf [file ae3c00625_si_001.pdf]

Supporting Information

Hybrid nanostructured compounds of Mo_2C on vertical graphene nanoflakes for a highly efficient hydrogen evolution reaction.

Stefanos Chaitoglou[1,2*], Roger Amade[1,2], Rogelio Ospina[3], Enric Bertran-Serra[1,2]

*Corresponding Author: Dr. Stefanos Chaitoglou

Contact email: stefanoschaitoglou@ub.edu

1. Department of Applied Physics, University of Barcelona, C/Martí i Franquès, 1, 08028 Barcelona, Catalunya, Spain

2. ENPHOCAMAT Group, Institute of Nanoscience and Nanotechnology (IN2UB), Universitat de Barcelona, C/Martí i Franquès, 1, 08028 Barcelona, Catalunya, Spain

3. Escuela de Física, Universidad Industrial de Santander, Carrera 27 calle 9 Ciudad Universitaria Bucaramanga, Colombia

Supplementary figures

1. JCPDS patterns of graphite, MoO2 and a-Mo_2C...S2
2. Linear sweep voltammetry curves of Mo on GNWs/Papyex ®...........................S5
3. Digital images of nanostructured electrodes.. S6
4. EDS mapping and SEM images of Mo_2C/ GNWs compounds S7
5. Raman spectra of bare GNWs prior and after annealing................................. S8
6. SEM Images and Raman spectra of Mo carbides on GNWs............................. S9

7. TEM image of Mo_2C particle…. …………………………………………………….. S10

8. XPS graph of C_{1s} peak of GNWs/ Papyex ® before and after Mo deposition……… S11

9. Deconvolution of C_{1s} peak of of GNWs/ Papyex ® before and after carburization. S12

10. XPS graph of Mo_{3d} peak before carburization ………………………………………S13

11. XPS graph of Mo_{3d} peak under varying carburization duration…………………… S14

12. Chronoamperometry test and electrode characterization………………….……….. S15

13. Raman spectrum of Mo_2C onn Papyex ®…………………………….………….. S16

14. SEM image of Mo_2C on Papyex ©………………………………………………… S17

15. CV graphs of Mo_2C compounds………….…………….………………………… S18

16. CV graphs and current desity-scan rate plots of bare GNWs …………………….… S1

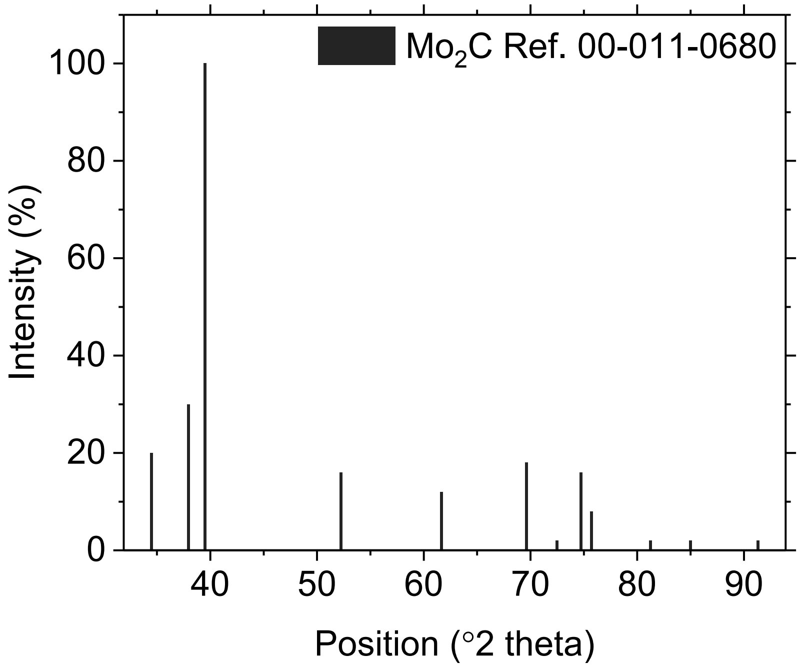

Peak list Mo_2C

No.	h	k	l	d [A]	2Theta[deg]	I [%]
1	1	0	0	2.60000	34.467	20.0
2	0	0	2	2.37000	37.934	30.0
3	1	0	1	2.28000	39.492	100.0
4	1	0	2	1.75000	52.230	16.0

5	1	1	0	1.50300	61.662	12.0
6	1	0	3	1.34900	69.642	18.0
7	2	0	0	1.30300	72.481	2.0
8	1	1	2	1.26900	74.748	16.0
9	2	0	1	1.25500	75.727	8.0
10	0	0	4	1.18300	81.255	2.0
11	2	0	2	1.14000	85.017	2.0
12	1	0	4	1.07700	91.324	2.0
13	2	0	3	1.00300	100.349	2.0
14	2	1	0	0.98400	103.040	2.0
15	2	1	1	0.96400	106.082	4.0
16	1	1	4	0.93000	111.845	4.0
17	2	1	2	0.90800	116.065	2.0
18	1	0	5	0.89000	119.881	2.0
19	2	0	4	0.87500	123.367	2.0
20	3	0	0	0.86800	125.107	2.0
21	2	1	3	0.83500	134.593	4.0
22	3	0	2	0.81500	141.872	2.0

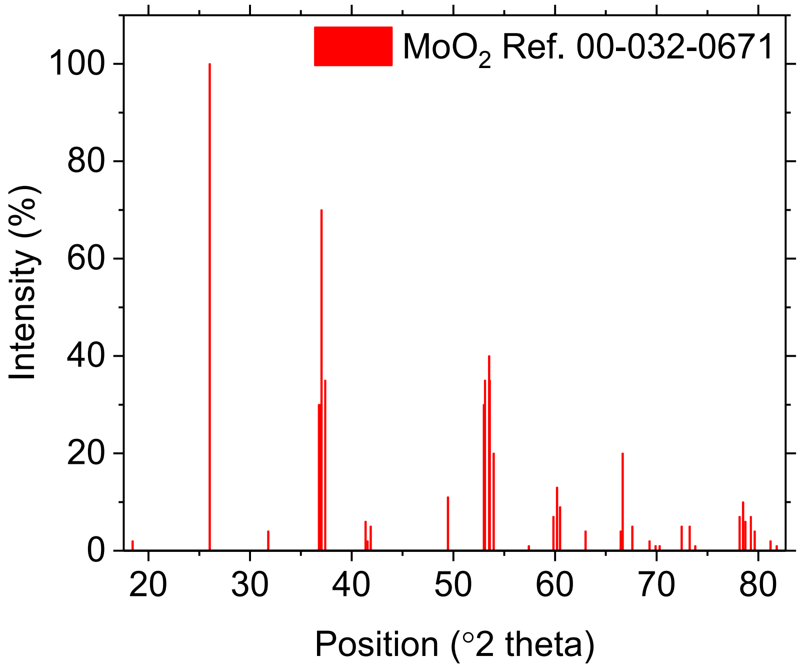

Peak list MoO_2

No.	h	k	l	d [A]	2Theta[deg]	I [%]
1	-1	0	1	4.80500	18.450	2.0
2	-1	1	1	3.42000	26.033	100.0
3	1	0	1	2.81300	31.785	4.0
4	2	0	0	2.44200	36.775	30.0
5	1	1	1	2.43700	36.853	30.0
6	-2	1	1	2.42600	37.026	70.0
7	-2	0	2	2.40300	37.393	35.0
8	2	1	0	2.18100	41.365	6.0

9	0	2	1	2.17100	41.564	2.0
10	-2	1	2	2.15600	41.867	5.0
11	-3	0	1	1.84100	49.469	11.0
12	2	1	1	1.72500	53.045	30.0
13	-2	2	0	1.72300	53.112	35.0
14	-3	1	2	1.71100	53.514	40.0
15	-2	2	2	1.70900	53.581	35.0
16	-2	1	3	1.69760	53.970	20.0
17	-3	0	3	1.60330	57.429	1.0
18	-3	1	0	1.54430	59.842	7.0
19	0	3	1	1.53600	60.198	13.0
20	0	1	3	1.52720	60.581	9.0
21	-3	2	1	1.46760	63.319	4.0
22	2	0	2	1.40570	66.458	4.0
23	-4	0	2	1.40190	66.661	20.0
24	-2	0	4	1.38450	67.611	5.0
25	3	0	1	1.35480	69.301	2.0
26	0	3	2	1.34480	69.891	1.0
27	-3	2	3	1.33810	70.293	1.0
28	-4	1	1	1.30330	72.461	5.0
29	-4	1	3	1.29120	73.250	5.0
30	-3	1	4	1.28290	73.802	1.0
31	4	0	0	1.22190	78.161	7.0
32	2	3	1	1.21750	78.498	10.0
33	1	3	2	1.21460	78.721	6.0
34	-2	3	3	1.20760	79.267	7.0
35	-2	2	4	1.20280	79.647	4.0
36	3	2	1	1.18370	81.197	2.0
37	1	2	3	1.17640	81.808	1.0
38	-3	3	0	1.14850	84.242	2.0
39	0	3	3	1.14140	84.889	4.0

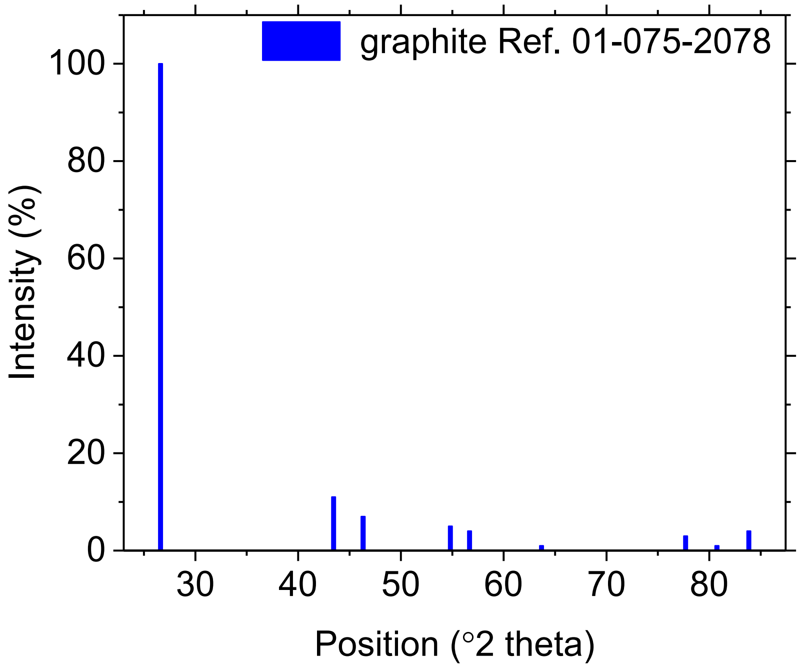

Peak list graphite

No.	h	k	l	d [A]	2Theta[deg]	I [%]
1	1	1	1	3.34701	26.611	100.0
2	0	1	0	2.08083	43.455	11.3
3	1	1	0	1.95849	46.322	7.2
4	2	2	2	1.67351	54.812	5.0
5	1	1	2	1.62279	56.677	4.0
6	2	2	1	1.46021	63.677	1.4
7	-1	1	0	1.22803	77.697	3.2
8	2	3	2	1.18926	80.739	1.0
9	1	2	0	1.15288	83.849	4.9
10	3	3	3	1.11567	87.329	0.7

Figure S1: Data of the JCPDS patterns of graphite (bottom), MoO_2 (middle) and a-Mo_2C (top).

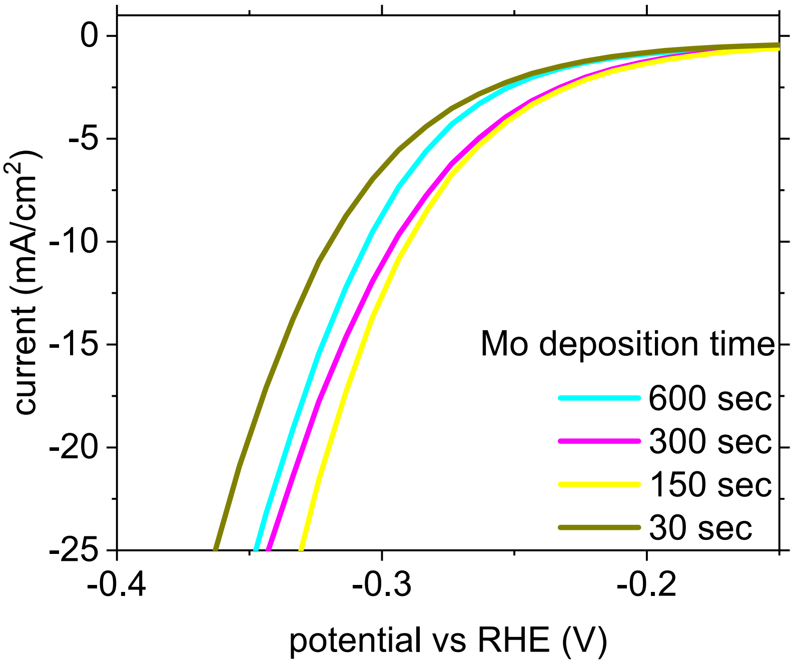

Figure S2: Linear sweel voltammetry curves of Mo deposited on GNWs/ Papyex ®, under various deposition times

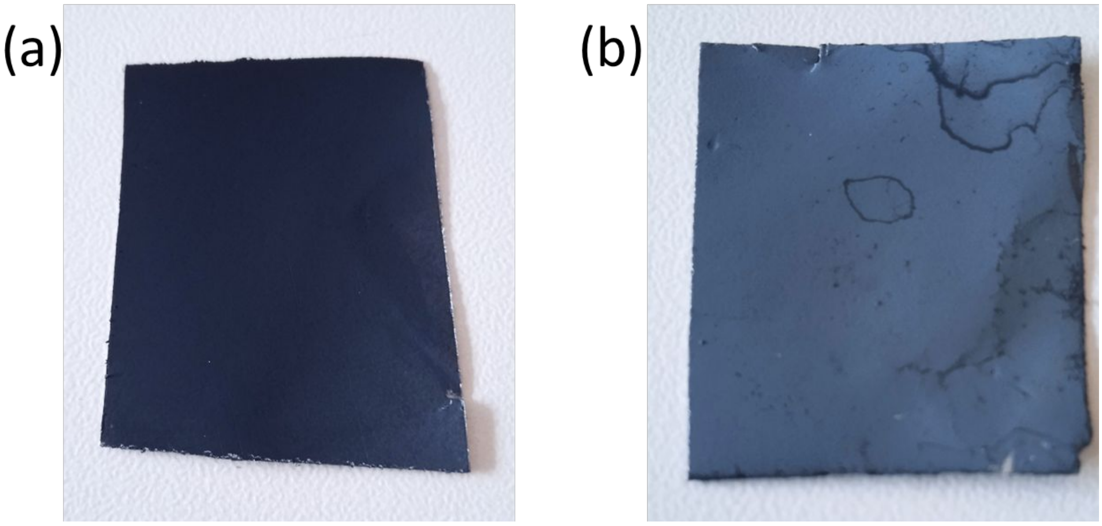

Figure S3: Digital image of a) GNWs on Papyex electrode and b) nanostructured Mo_2C on GNWs electrode.

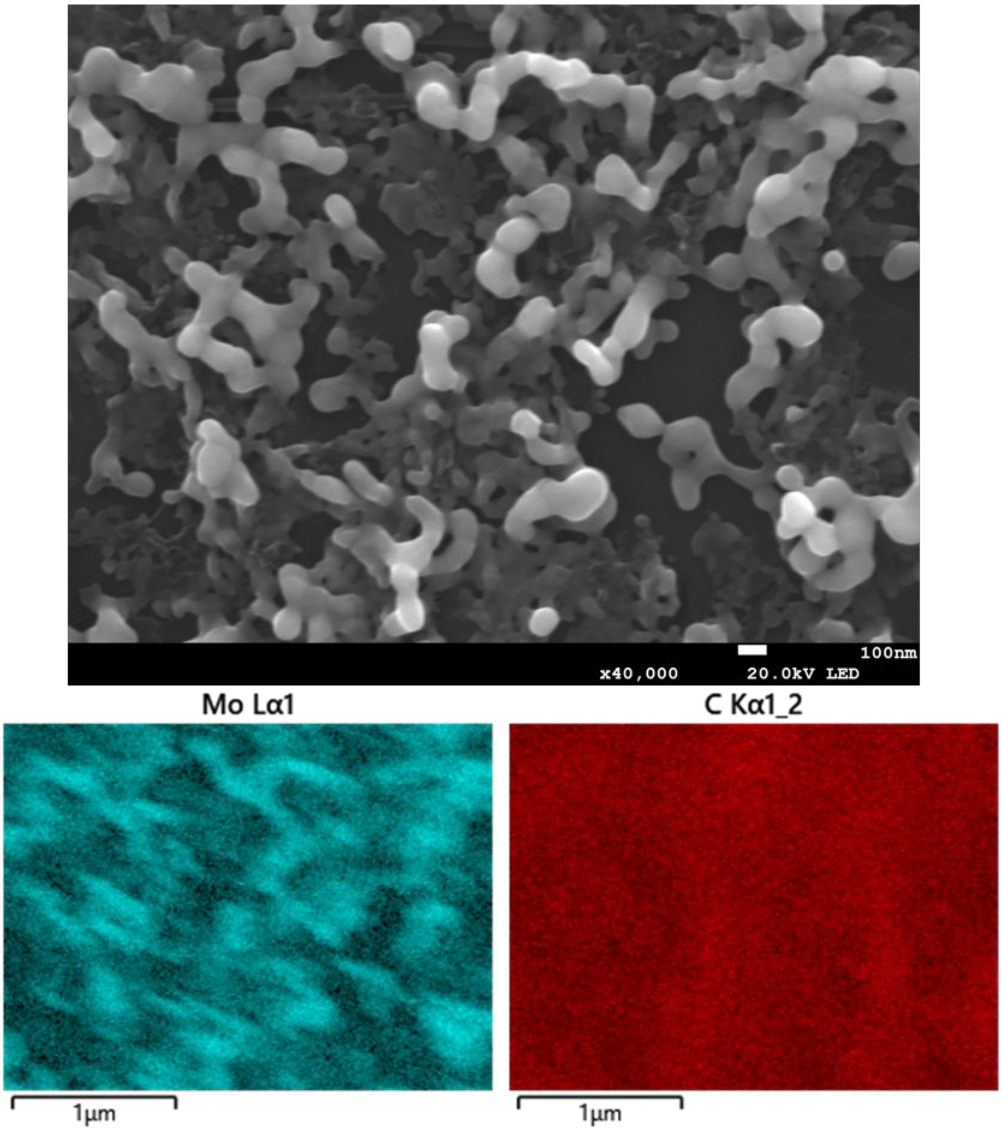
100nm
x40,000
20.0kV LED
Mo Lα1
C Kα1_2
1μm
1μm

Figure S4: Mo and C EDS mapping and SEM image of Mo_2C/GNWs prepared after 15 min of annealing times.

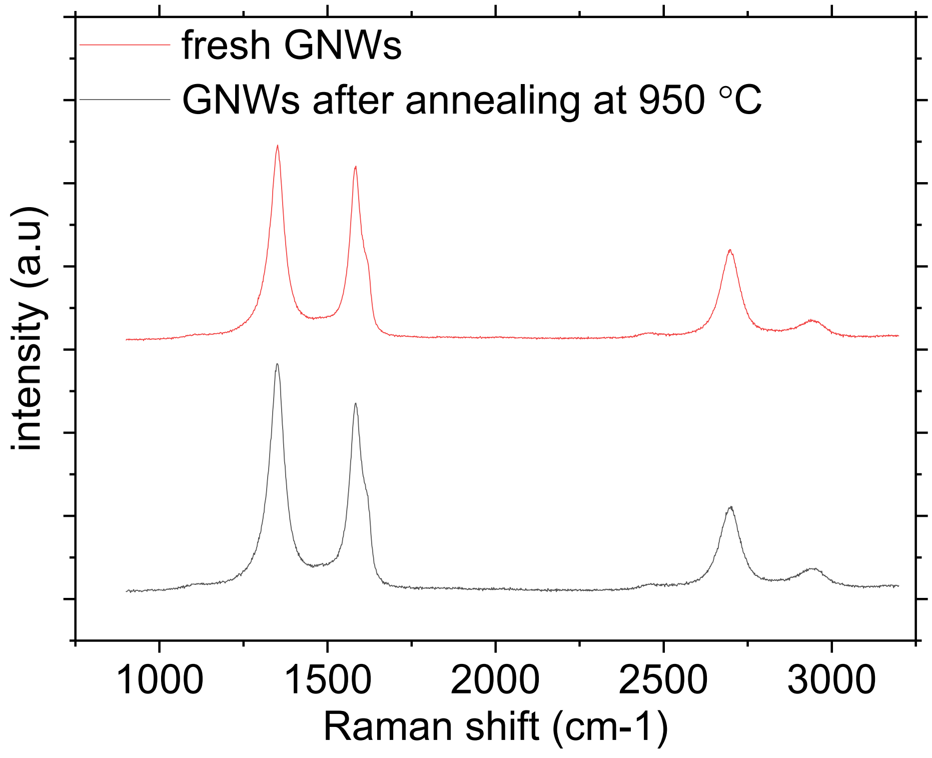

Figure S5: Raman spectra of bare GNWs, fresh (red line) and after thermal annealing at 950 ˚C (black line) in Ar atmosphere, showing luck of any structural alteration.

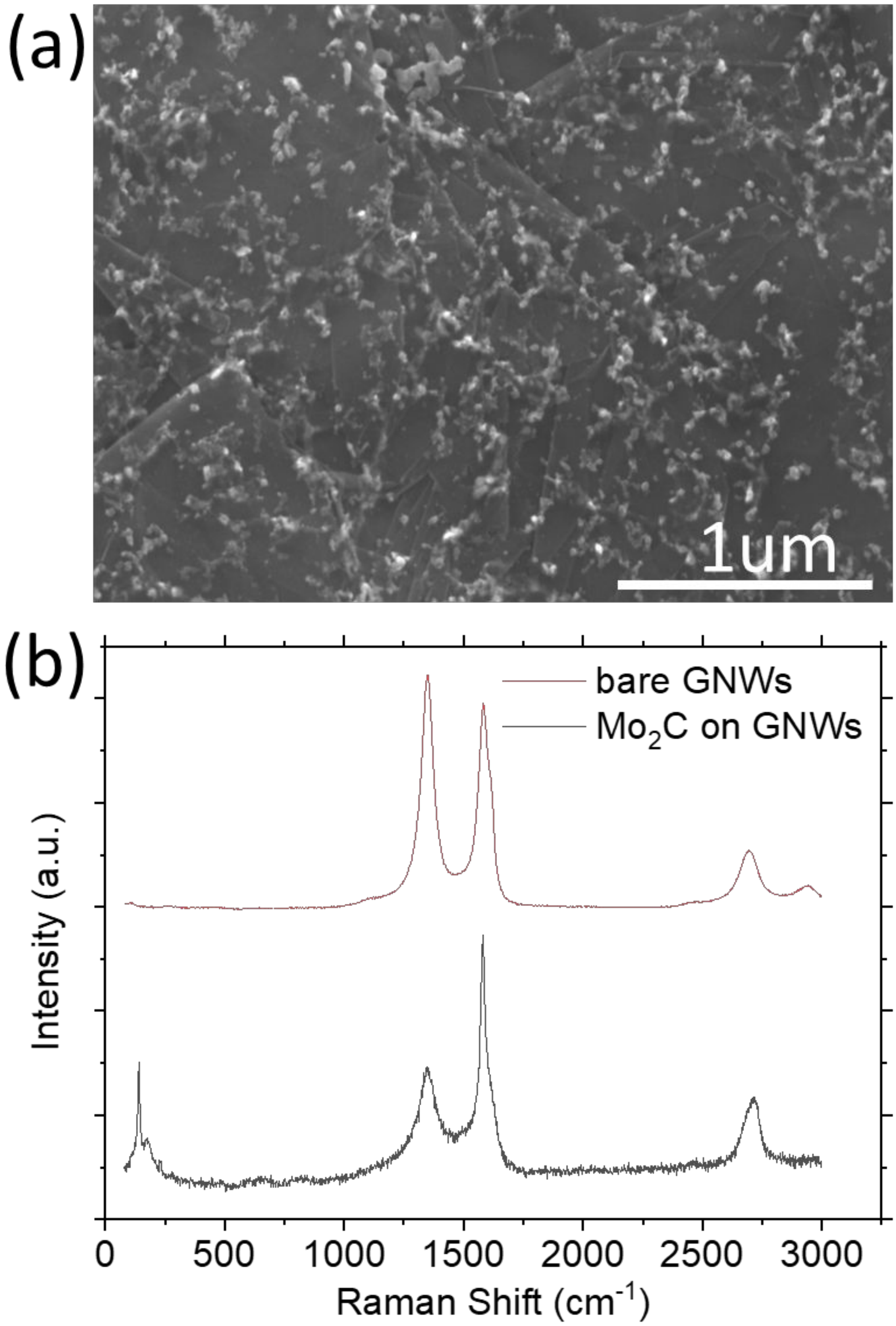

Figure S6: a) SEM image of Mo_2C deposited on GNWs/Papyex ©, after carburization in CH_4 atmosphere. Partial etching of the GNWs is observed. b) Raman spectra of the sample before

and after Mo_2C deposition reveal the deterioration in the crystal quality of the GNWs, as a result of the carburization process in CH_4 atmosphere.

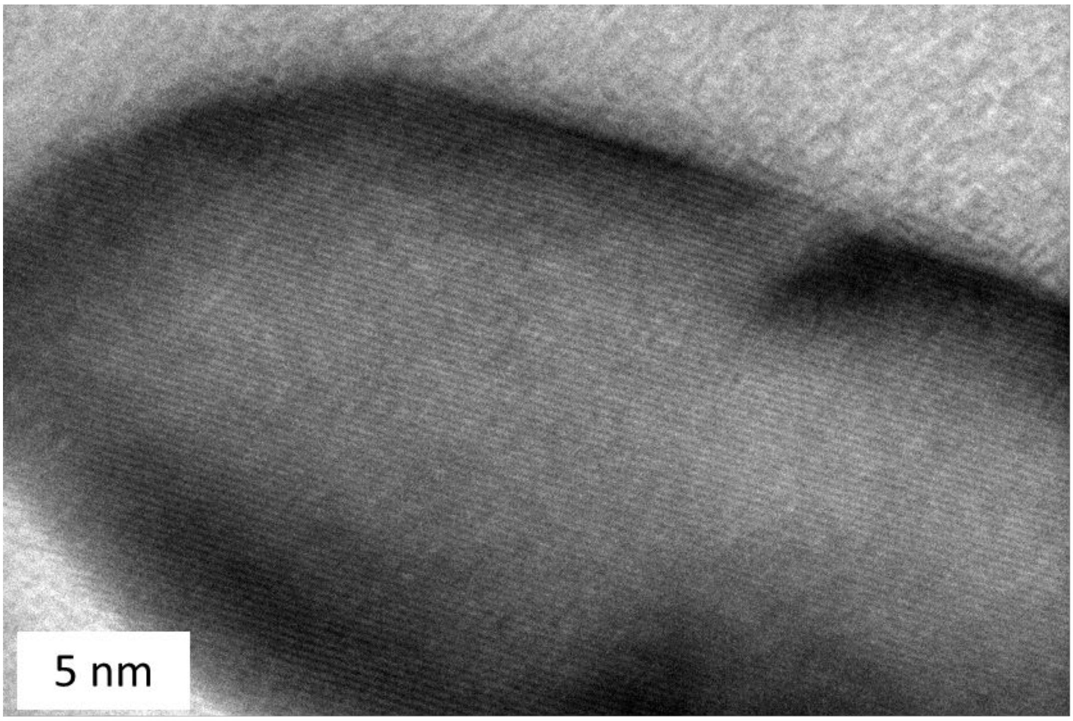

Figure S7: TEM image of a single crystal Mo_2C particle.

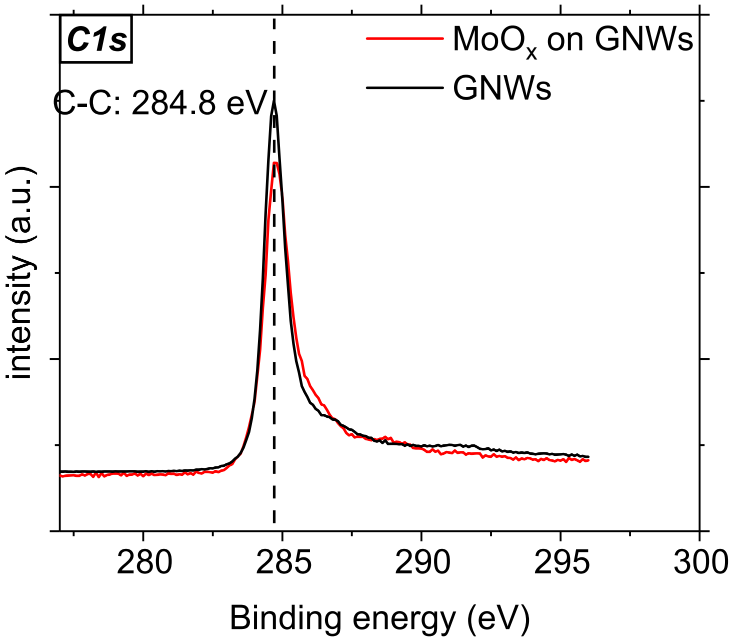

Figure S8: XPS graph of C_{1s} peak of GNWs/ Papyex and Mo deposited on GNWs on Papyex , revealing absence of Mo-C interaction.

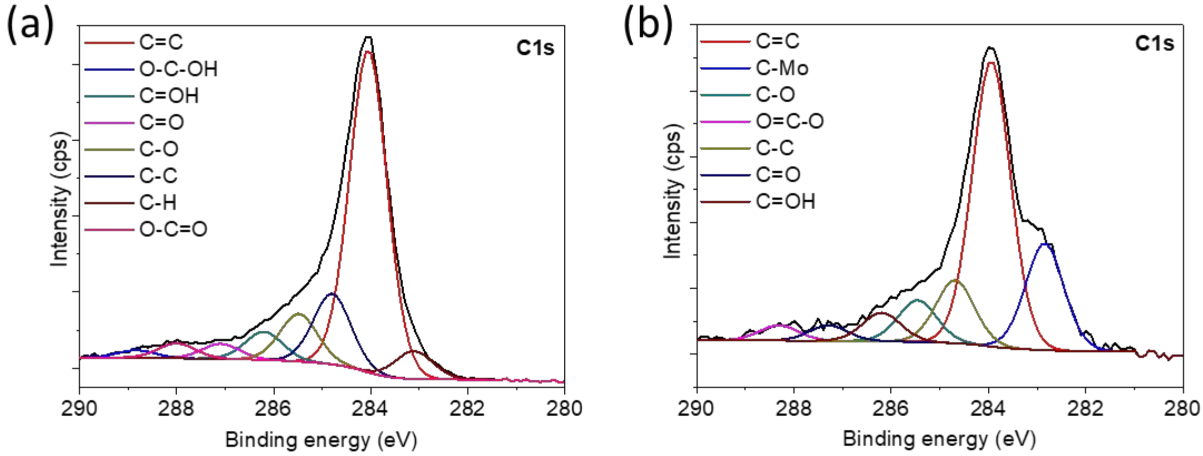

Figure S9: XPS graph of C_{1s} peak a) before and b) after carburization.

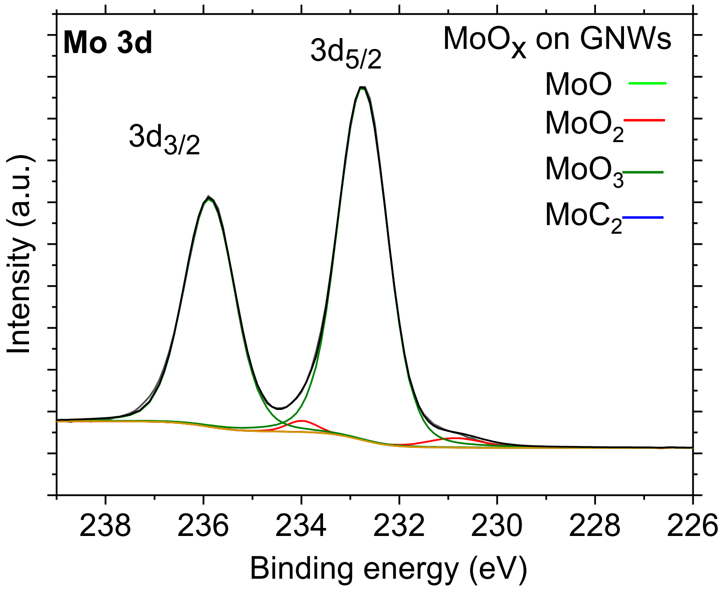

Figure S10: XPS graph of Mo3d peak before carburization.

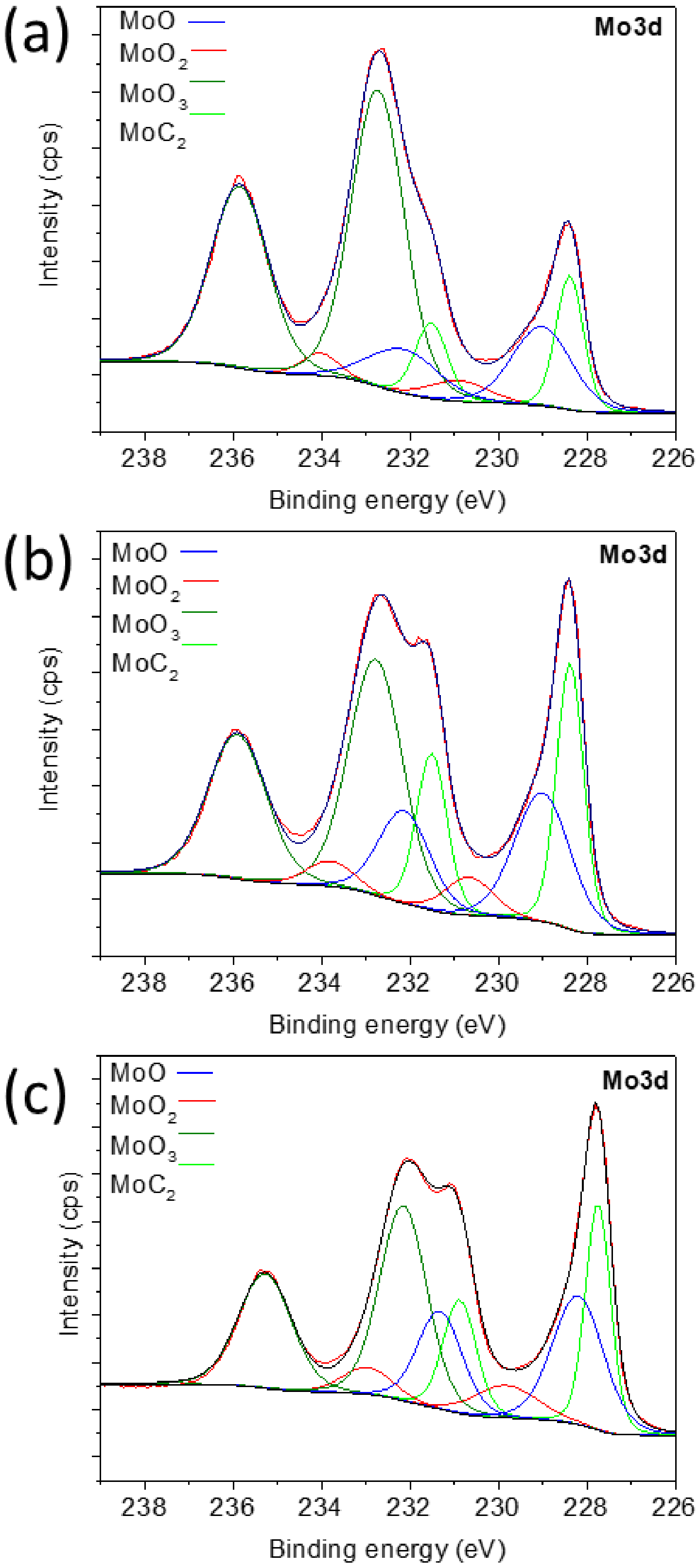

Figure S11: XPS graph of Mo_{3d} peak after 4’ (a), 8’ (b) and 15’ (c) of carburization duration.

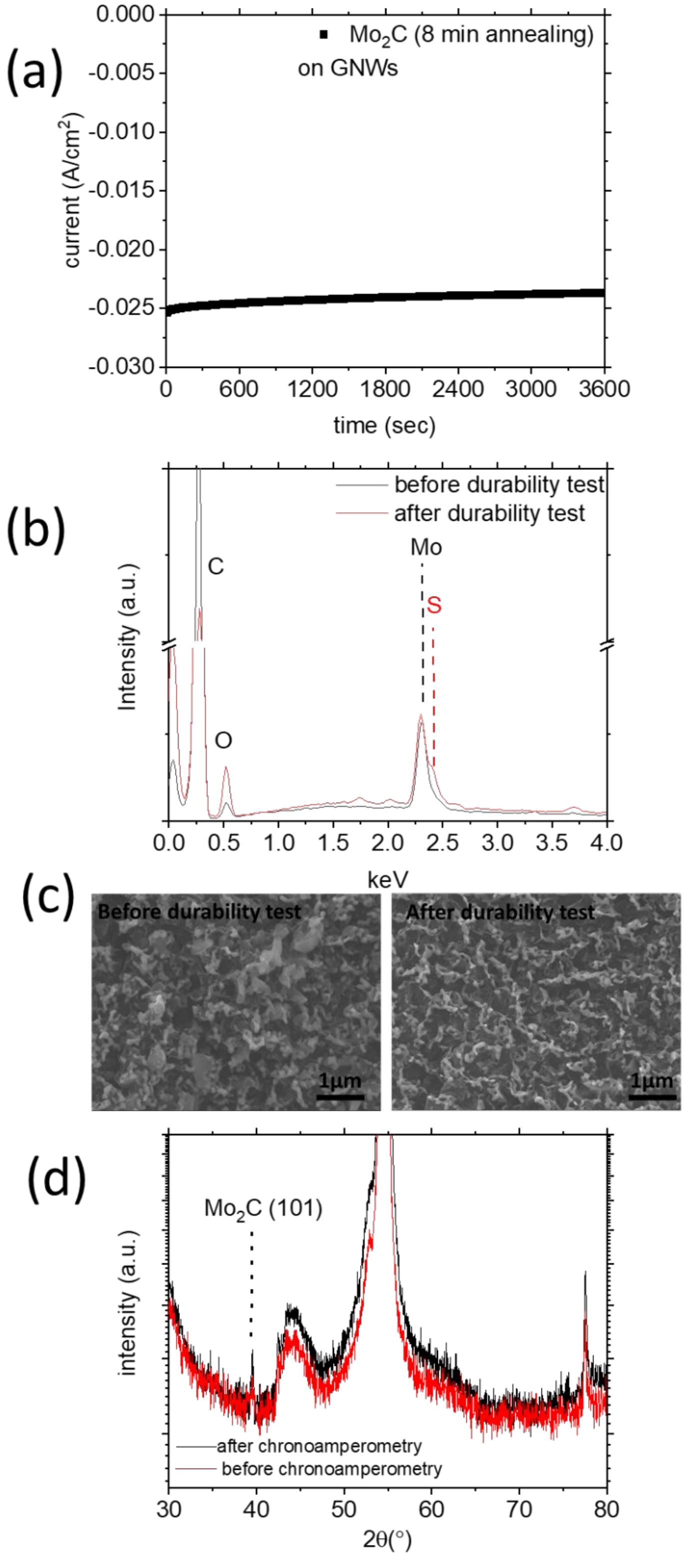

Figure S12: a) Chronoamperometry test during 1 h under 200 mV of constant bias vs RHE. b) EDS analysis, c) SEM images and d) XRD analysis, of the electrode before and after the test

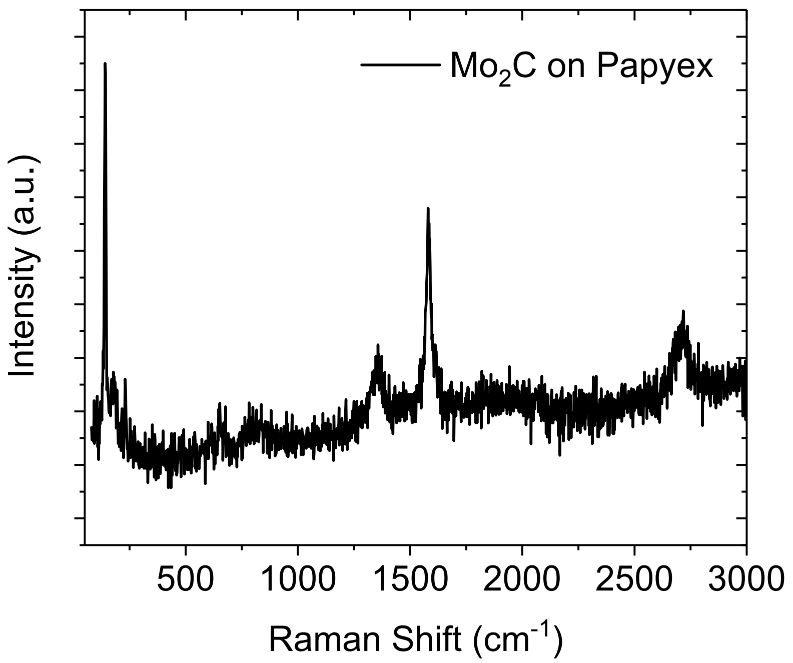

Figure S13: Raman spectrum of Mo_2C on Papyex substrate.

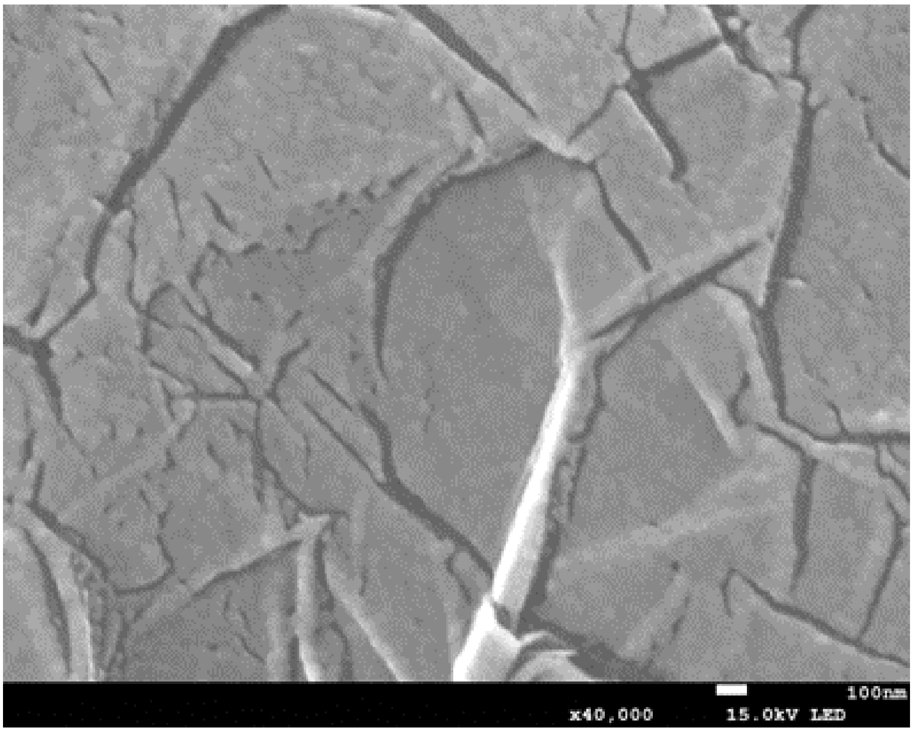

Figure S14: SEM image of a Mo_2C film formed on a Papyex substrate, after carburization of sputtered Mo in a CH_4 atmosphere.

.

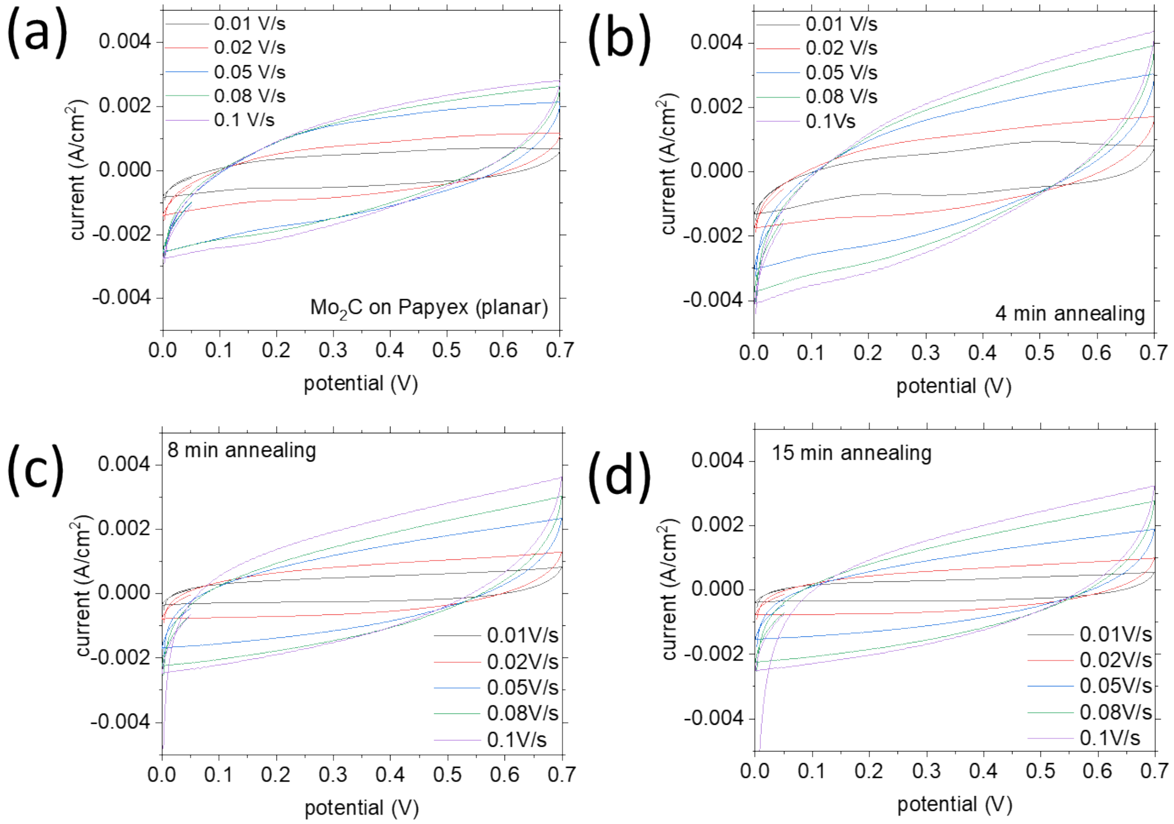

Figure S15: CV graphs of Mo carbide deposited on a) Papyex and GNWs after b) 4’, c) 8’ and c) 15’ of annealing duration, obtained under varying scan rates

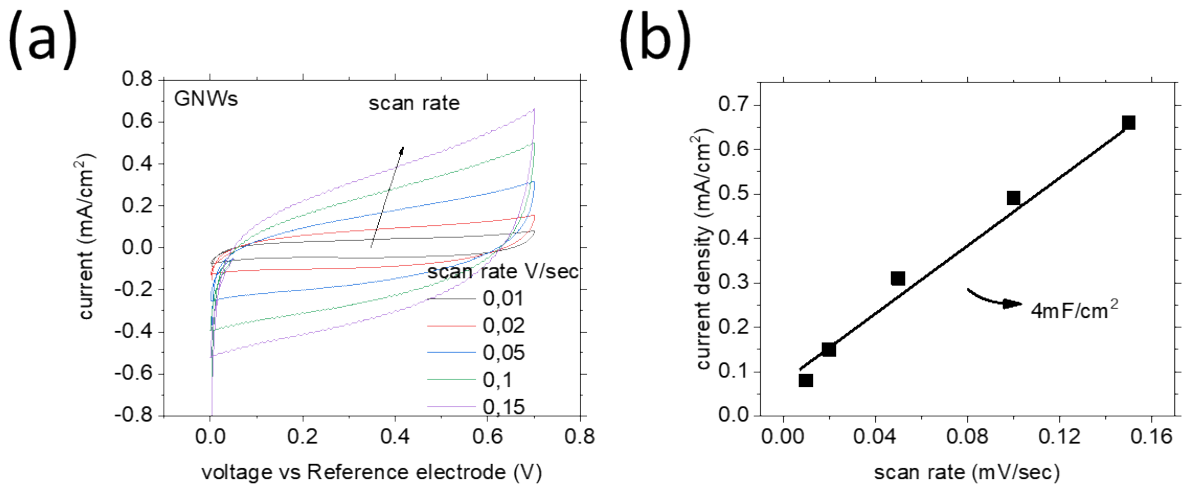

Figure S16: a) CV graphs and b) current density-scan rate plots of bare GNWs electrodes.
